# Analysis of spatial and temporal patterns of aboveground net primary productivity in the Eurasian steppe region from 1982 to 2013

**DOI:** 10.1002/ece3.3027

**Published:** 2017-06-06

**Authors:** Cuicui Jiao, Guirui Yu, Jianping Ge, Xi Chen, Chi Zhang, Nianpeng He, Zhi Chen, Zhongmin Hu

**Affiliations:** ^1^ Synthesis Research Center of Chinese Ecosystem Research Network Key Laboratory of Ecosystem Network Observation and Modeling Institute of Geographic Sciences and Natural Resources Research Chinese Academy of Sciences Beijing China; ^2^ University of Chinese Academy of Sciences Beijing China; ^3^ College of Resources and Environment University of Chinese Academy of Sciences Beijing China; ^4^ College of Life Sciences Beijing Normal University Beijing China; ^5^ State Key Laboratory of Desert and Oasis Ecology Xinjiang Institute of Ecology and Geography Chinese Academy of Sciences Urumqi Xinjiang China; ^6^ CAS Research Center for Ecology and Environment of Central Asia Urumqi Xinjiang China

**Keywords:** aboveground net primary productivity, composite period, Eurasian steppe region, normalized difference vegetation index, spatial patterns, temporal dynamics

## Abstract

To explore the importance of the Eurasian steppe region (EASR) in global carbon cycling, we analyzed the spatiotemporal dynamics of the aboveground net primary productivity (ANPP) of the entire EASR from 1982 to 2013. The ANPP in the EASR was estimated from the Integrated ANPP_NDVI_ model, which is an empirical model developed based on field‐observed ANPP and long‐term normalized difference vegetation index (NDVI) data. The optimal composite period of NDVI data was identified by considering spatial heterogeneities across the study area in the Integrated ANPP_NDVI_ model. EASR's ANPP had apparent zonal patterns along hydrothermal gradients, and the mean annual value was 43.78 g C m^−2^ yr^−1^, which was lower than the global grasslands average. Compared to other important natural grasslands, EASR's ANPP was lower than the North American, South American, and African grasslands. The total aboveground net primary productivity (TANPP) was found to be 378.97 Tg C yr^−1^, which accounted for 8.18%–36.03% of the TANPP for all grasslands. In addition, EASR's TANPP was higher than that of the grasslands in North America, South America, and Africa. The EASR's TANPP increased in a fluctuating manner throughout the entire period of 1982–2013. The increasing trend was greater than that for North American and South American and was lower than that for African grasslands over the same period. The years 1995 and 2007 were two turning points at which trends in EASR's TANPP significantly changed. Our analysis demonstrated that the EASR has been playing a substantial and progressively more important role in global carbon sequestration. In addition, in the development of empirical NDVI‐based ANPP models, the early–middle growing season averaged NDVI, the middle–late growing season averaged NDVI and the annual maximum NDVI are recommended for use for semi‐humid regions, semi‐arid regions, and desert vegetation in semi‐arid regions, respectively.

## 1 INTRODUCTION

Aboveground net primary productivity (ANPP) represents the major input of nutrition and energy into ecosystems, and it is an integral indicator of ecosystem functions (McNaughton, Oesterheld, Frank, & Williams, 1989). ANPP is one of the main components of the carbon cycle and one of the most important and fundamental fluxes that reflect carbon sinks/sources of ecosystems (Scurlock, Cramer, Olson, Parton, & Prince, 1999). ANPP can indicate the growth status of vegetation, for which variations over time reflect the response of ecosystems to climate change (Roy, Mooney, & Saugier, 2001). In addition, ANPP is a good index of potential economic production (food, fuel, fiber) (Scurlock et al., 1999). The spatiotemporal dynamics of ANPP have been a key research topic of Global Change and Terrestrial Ecosystems (GCTE) (Fang et al., 2003; Steffen et al., 1998).

Globally, grasslands are one of the most widespread biomes, and they are known as the prairie in North America, the pampas in South America, the veld in South Africa, the steppe in Eurasia, and the savanna in Africa and Australia (Woodward, 2008). These grasslands account for approximately 20% of the world's land surface (Lieth, 1978). From a perspective of carbon cycles of ecosystems, grasslands likely contribute an annual carbon sink of up to ~0.5 Pg C (Scurlock & Hall, 1998), and these systems amount to approximately 18% of the total current global terrestrial carbon sink (Canadell et al., 2007), playing a key role in balancing the concentration of global atmospheric greenhouses gases through carbon sequestration (Lauenroth, 1979; O'Mara, 2012). From an applied perspective, grasslands significantly contribute to resources needed for human activities by providing food for herbivores (O'Mara, 2012; Scurlock et al., 1999). Therefore, examining spatiotemporal ANPP patterns in grasslands is critical to understanding terrestrial carbon sequestration and fundamental for determining the sustainable use of grassland resources (O'Mara, 2012; White, Murray, & Rohweder, 2000).

Evaluations of ANPP in grasslands at a regional or global scale have suggested that the ANPP of grasslands exhibits obvious spatial variations (Bao et al., 2016; Eisfelder, Klein, Niklaus, & Kuenzer, 2014; Irisarri, Oesterheld, Paruelo, & Texeira, 2012; Sala, Parton, Joyce, & Lauenroth, 1988; Xia et al., 2014; Zhang et al., 2014). Estimates of total aboveground net primary productivity (TANPP) in global grasslands vary from 1423 Tg C yr^−1^ to 4635 Tg C yr^−1^ (Bazilevich, Rodin, & Rozov, 1971; Parton et al., 1995; Whittaker & Likens, 1975; Xia et al., 2014). In addition, the TANPP of global grasslands displays a significant increasing trend for the past three decades (Xia et al., 2014). North American grasslands (Goward & Dye, 1987; Lauenroth, 1979; Xia et al., 2014), South American grasslands (Xia et al., 2014), and African grasslands (Xia et al., 2014), respectively, account for 9%–16%, 3%–14% and 4% ‐13% of the TANPP for global grasslands. In the past three decades, TANPP showed an obvious increasing trend in North American and African grasslands as well as a slight downward trend in South American grasslands (Xia et al., 2014).

**Figure 1:**
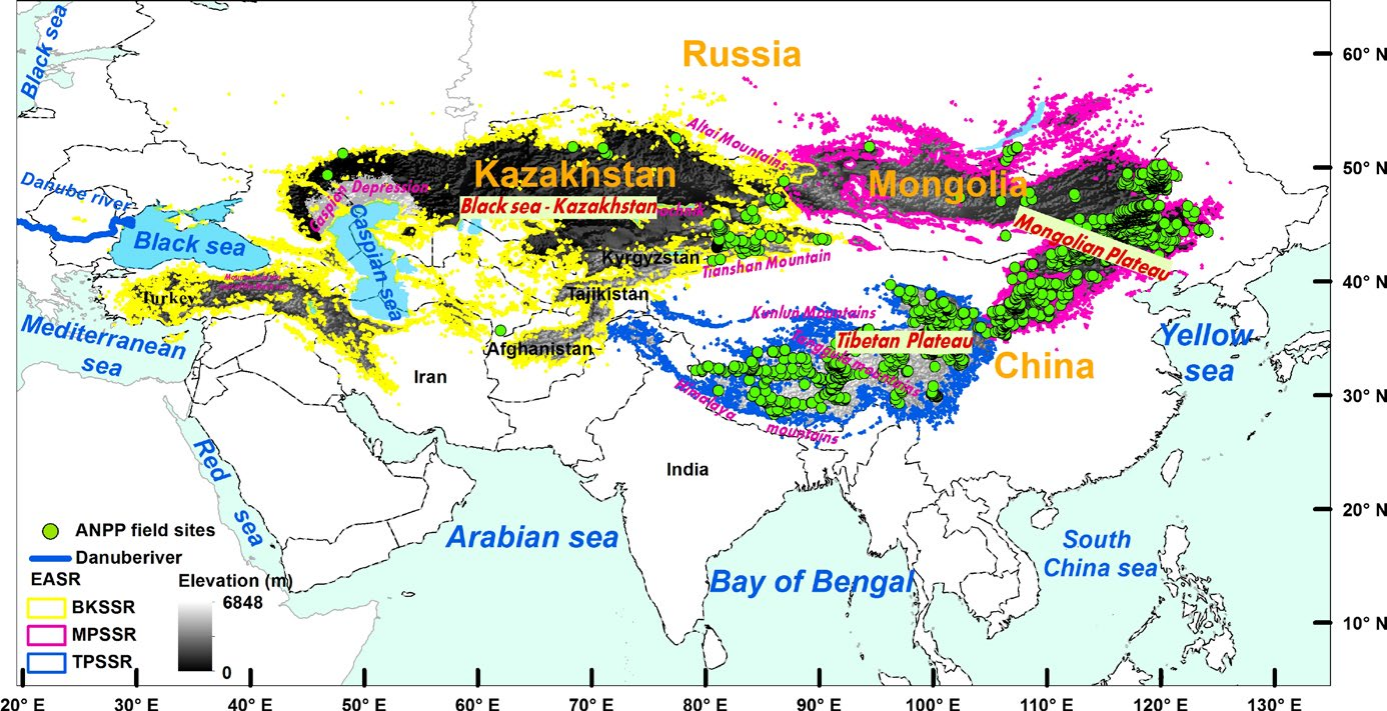
The geographic extent of the Eurasian steppe region and the spatial distribution of ANPP field sites. ANPP denotes the aboveground net primary productivity per year and per square meter. EASR denotes the Eurasian steppe region, BKSSR denotes the Black Sea–Kazakhstan steppe subregion, MPSSR denotes the Mongolian Plateau steppe subregion, and TPSSR denotes the Tibetan Plateau alpine steppe subregion

The Eurasian steppe region (EASR), which is the largest continuous grassland biome worldwide, plays an important role in global grasslands, as do North American, South American, and African grasslands (Woodward, 2008). The EASR, which is located in northern mid‐latitudes, is influenced by monsoon, continental, and Mediterranean climates, and it is sensitive to global environmental change (Figure 1). Studies on the spatiotemporal dynamics of the ANPP of the EASR mainly focus on typical geographical units within it, such as on the Inner Mongolian temperate grasslands (Ma, Fang, Yang, & Mohammat, 2010; Ma, Liu et al., 2010; Ma, Yang, He, Hui, & Fang, 2008) and the Tibetan Plateau alpine grasslands (Zhang et al., 2014). In recent years, some studies have also analyzed the spatiotemporal dynamics of the ANPP in the grassland areas of Mongolia and Kazakhstan at a national scale (Bao et al., 2016; Eisfelder et al., 2014). However, studies on the spatiotemporal dynamics of ANPP have not yet been specifically reported for the entire EASR. There is thus a knowledge gap regarding the spatiotemporal dynamics of the ANPP for the entire EASR and the roles that the EASR plays in the global carbon budget. Therefore, studying the spatiotemporal patterns of the EASR's ANPP will further our understanding of carbon cycling mechanisms in grassland ecosystems and will prove central to the assessment of global carbon budgets.

The Normalized Difference Vegetation Index (NDVI) is the normalized reflectance difference between the satellite near‐infrared band and the visible red band (Rouse, Haas, Schell, & Deering, 1974; Tucker, 1979). The NDVI represents the photosynthetic potential of the vegetation canopy and is extensively used in ecosystem monitoring (An, Price, & Blair, 2013; Box, Holben, & Kalb, 1989; Gu, Wylie, & Bliss, 2013; Gu, Wylie, & Howard, 2015; Hobbs, 1995; Paruelo, Epstein, Lauenroth, & Burke, 1997; Rouse et al., 1974; Tucker, 1979). Previous studies have shown strongly positive relationships between the NDVI and ANPP in grasslands. Developing an NDVI‐based empirical remote sensing inversion model for ANPP estimation involves obtaining NDVI values over a specified time period (composite periods of NDVI data) (Reed et al., 1996). Composite periods of NDVI data may affect the accuracy of empirical NDVI‐based annual ANPP estimation models. The optimal period of the NDVI composite varies according to regional climatic conditions (An et al., 2013; Mkhabela, Bullock, Raj, Wang, & Yang, 2011) and vegetation type (Jin et al., 2014; Mao, Wang, Li, & Ma, 2014).

Therefore, in this study, we attempted to achieve the following objectives based on field‐observed ANPP and long‐term NDVI time‐series data: (1) to identify the best composite period of NDVI data for developing a robust annual ANPP estimation model designed for the entire EASR, (2) to evaluate the ANPP of the entire EASR, and (3) to explore the temporal dynamics of the EASR's TANPP and further discuss the role of the EASR in the global carbon budget.

## 2 MATERIALS AND METHODS

### 2.1 Study region

The Eurasian steppe region (EASR) (Figure 1) in the northern mid‐latitudes extends over approximately 110 longitudinal units from the grassy plains at the mouth of the Danube River in the west; across Russia, Kazakhstan, and Mongolia to the Songliao Plain in China to the east; and to the Himalayas in China to the southwest (Woodward, 2008). The EASR is the largest continuous grassland biome in the world, covering an area of 8.65 million km^2^, and the region is preserved relatively well (Woodward, 2008). The EASR is influenced by the Mediterranean climate, by the southwest monsoon of the Indian Ocean, by the East Asian monsoon and by the westerlies. Multiyear mean annual precipitation levels in the region vary from 60 mm to 1,100 mm (Appendix S1), and the mean annual temperatures (MATs) range from −9 to 20°C (Appendix S1) across the entire EASR, with rainy and hot conditions characterizing this period (Appendix S2).

Throughout the EASR, natural vegetation mainly includes meadows, meadow steppes, typical steppes, desert steppes, alpine steppes, and alpine meadows (Appendix S3, Olson et al., 2001; Editorial Committee of Vegetation Map of China Chinese Academy of Sciences, 2007; Woodward, 2008; Bao et al., 2016). The dominant grasses include perennial bunchgrasses, and constructive species belong to the *Stipa* genus, which refers to species controlling the structure and function of the ecosystem while their amount being not always maximum in the ecosystem (Woodward, 2008; Zhou, 1980). Chestnut soil is the main soil type (Woodward, 2008). Phytogeographically, the EASR can be divided into three subregions (Figure 1): the Black Sea–Kazakhstan steppe subregion (BKSSR), the Mongolian Plateau steppe subregion (MPSSR), and the Tibetan Plateau alpine steppe subregion (TPSSR) (Li, 1979; Wu, 1979; Лaвpeнкo, 1959).

It is important to note that the geographical extent of the EASR has not yet been clearly defined, so we identify the regional extents of the EASR and of its three subregions based on Moderate Resolution Imaging Spectroradiometer (MODIS) Land cover data according to descriptions of the EASR provided in Лaвpeнкo (1959), Wu (1979), Li (1979) and Woodward (2008).

### 2.2 Data collection

#### 2.2.1 Field‐observed ANPP data

In this study, field‐observed ANPP (ANPP_obs_) was estimated as the peak aboveground biomass for harvesting during the growing season (from April to October of the year). These data were primarily obtained from three sources: (1) 1015 ANPP field observations from 206 publications (a list of the data sources can be found in Appendix S11) cited in the Web of Science (www.Webofknowledge.com) and China National Knowledge Infrastructure (http://epub.cnki.net), (2) 7 ANPP field observations from the Class A dataset of the global ANPP database of the Oak Ridge National Laboratory Distributed Active Archive Center (https://daac.ornl.gov/cgi-bin/dataset_lister.pl?p=13#grassland_anchor) and (3) 809 ANPP field observations provided by the researchers of this study. In total, 1990 site‐year ANPP observations from 1831 field sites were initially collected for the entire EASR over the past three decades (1982–2013).

Before carrying out our analysis, we completed four tasks to eliminate unsuitable field‐observed ANPP data: (1) we excluded observations missing site‐description metadata (e.g., latitude or longitude), (2) we excluded observations without specific sampling times, (3) we excluded observations in ecotones of grasslands and other systems according to the Moderate Resolution Imaging Spectroradiometer (MODIS) Land cover product (MCD12C1) (https://lpdaac.usgs.gov/dataset_discovery/modis/Modisproducts_table/mcd12c1), and (4) we excluded observations with ANPP outliers (falling outside a range of mean ± 3 standard deviations).

Thus, field ANPP observations of 1717 site years from 1539 field sites were examined in this study. These ANPP field sites spanned 28°N to 53°N in latitude, 36°E to 125°E in longitude, and 20 m to 5600 m in elevation (Figure 1). In addition, the sampling time span ranged from 1982 to 2013 (Appendix S4).

It is worth noting that ANPP (g C m^−2^ yr^−1^) in this study denotes the aboveground net primary productivity per year and per square meter, and TANPP (Tg C yr^−1^) denotes the regional total aboveground net primary productivity level per year. Field data reported in dry matter form (g m^−2^ yr^−1^) from previous studies were converted into units of C using a conversion factor of 0.45 (Lieth & Whittaker, 1975).

#### 2.2.2 Remote sensing data

##### Long‐term NDVI time‐series data

The biweekly NDVI data for 1982 to 2013 used in this study were obtained from third‐generation NDVI (NDVI3 g) datasets produced through the Global Inventory Modeling and Mapping Studies (GIMMS). GIMMS NDVI3 g data (http://ecocast.arc.nasa.gov/data/pub/gimms/3g.v0/) were obtained at a spatial resolution of .083° by applying the 15‐day maximum‐value composition (MVC) technique (Holben, 1986) to observations generated by Advanced Very High Resolution Radiometers (AVHRRs) aboard National Oceanic and Atmospheric Administration (NOAA) satellites (Tucker et al., 2005; Zhu et al., 2013). GIMMS NDVI3 g data were corrected for sensor degradation, intersensor differences, cloud cover, solar zenith angle, and viewing angle effects resulting from satellite drift as well as the presence of volcanic aerosols. These data were widely used to monitor long‐term vegetation activation trends (Piao et al., 2005; Wu & Liu, 2013).

##### Land cover data

The land cover data used in this study were collected from the Land Cover Type Climate Modelling Grid (CMG) product (MCD12C1) in 2012. The MCD12C1 (LP DAAC; https://lpdaac.usgs.gov) at a spatial resolution of .05° was derived from observations covering one year of Terra and Aqua MODIS data collected from Earth Observation Systems (EOS) satellites. The MCD12C1 included three classification schemes: the International Geosphere Biosphere Programme (IGBP) global vegetation classification scheme, the University of Maryland (UMD) scheme, and the MODIS‐derived LAI/FPAR scheme (Friedl et al., 2010). The land cover data used in this study were based on the IGBP global vegetation classification scheme.

#### 2.2.3 Climatic data

Monthly climatic data (including air temperature and precipitation data) were derived from meteorological data with a spatial resolution of 0.5° stored at the University of East Anglia's Climate Research Unit (CRU TS 3.23) (https://crudata.uea.ac.uk/cru/data/hrg/cru_ts_3.23/). CRU TS 3.23 climate data were obtained through an interpolation of average monthly climate data from weather stations (Harris, Jones, Osborn, & Lister, 2014; Mitchell & Jones, 2005; New, Hulme, & Jones, 1999, 2000; New, Lister, Hulme, & Makin, 2002).

### 2.3 Data analysis

#### 2.3.1 ANPP estimation model development

The best ANPP estimation model for the entire EASR was accomplished through following three steps:

##### Data preprocessing

Before developing the ANPP estimation model, we completed the following three data preprocessing tasks.

1. First, monthly NDVI series data were generated by applying MVC (Equation (1), Holben, 1986) to biweekly NDVI series data.

(1) $${MNDVI}_{i}=Max\left({NDVI}_{ia},\;{NDVI}_{ib}\right),$$

where *i* is the month from month 1 to month 12, *MNDVI*_*i*_ is the maximum of two NDVI images available for month *i*, and *NDVI*_*ia*_ and *NDVI*_*ib*_ are NDVI images of the first and second halves of month *i*, respectively.

- The optimal composite period of monthly NDVI data for generating annual NDVI values for ANPP estimation varies with the climate and vegetation conditions in the study area (An et al., 2013; Mkhabela et al., 2011). Therefore, 13 different annual NDVI values were obtained by calculating the annual maximum NDVI and 12 different averaged NDVI values of various composite periods for April to October. Theses 12 different periods were identified by changing the start and end date of the monthly NDVI composite periods (Appendices S5 and S6).

- According to the geographical locations and the corresponding sampling year of field‐observed ANPP, we extracted 13 annual NDVI values for each ANPP field site. In turn, we generated a dataset in which every record included field‐observed data and 13 corresponding annual NDVI values. Approximately 75% of the field‐observed ANPP data were randomly selected to develop the ANPP estimation model, and the remaining 25% were used to validate the model. Given that NDVI images of sparsely vegetated areas can be affected by the spectral characteristics of soils, we only analyzed grasslands with an annual maximum NDVI value of > 0.1 in this study.

##### Model development

Based on the preprocessed data listed above, we developed ANPP estimation models for the entire EASR through two different schemes: the Entirety Overall Scheme and the Subregions Integrated Scheme. Then, the best ANPP estimation model specific to the entire EASR was obtained by selecting the model with higher validation accuracy. In the Entirety Overall Scheme, spatial heterogeneities across the EASR were not considered in the development of the ANPP estimation model. Thus, NDVI‐based variables for ANPP estimation were considered universal for the entire EASR in this scheme. By contrast, the ANPP estimation model developed in the Subregions Integrated Scheme considered spatial heterogeneities between three subregions of the EASR.

##### Model validation and optimization

The best ANPP estimation model (the Overall ANPP_NDVI_ model) for the entire EASR was identified through two phases in the Entirety Overall Scheme: (1) 52 regression models, including linear, exponential, power, and logarithmic models, were developed for field‐observed ANPP data and 13 annual NDVI values for the entire EASR, and (2) the performance of these models was assessed using the coefficient of determination (*R* ^2^, Equation 2) and the root mean square error (*RMSE*, Equation 3). The model that met the maximum *R* ^2^ and minimum *RMSE* criteria of the 52 regression models was regarded as the optimal ANPP estimation model for the entire EASR. Finally, the corresponding period was considered the optimal composite period of the NDVI for ANPP estimation in the EASR.

(2) $$R^{2}={\left\{\frac{\sum_{t=1}^{n}\left[{\left(ANPP_{obs}\right)}_{t}\;-\;\bar{\left(ANPP_{obs}\right)}\right]\left[{\left(ANPP{}_{mod}\right)}_{t}\;-\;\bar{\left(ANPP_{mod}\right)}\right]}{\sqrt{\sum_{t=1}^{n}{\left[{\left(ANPP_{obs}\right)}_{t}\;-\;\bar{\left(ANPP_{obs}\right)}\right]}^{2}\sum_{t=1}^{n}{\left[{\left(ANPP_{mod}\right)}_{t}\;-\;\bar{\left(ANPP_{mod}\right)}\right]}^{2}}}\right\}}^{2}$$

(3) $$RMSE\;=\;\sqrt{\frac{\sum_{t=1}^{n}{\left[{\left(ANPP_{obs}\right)}_{t}-{\left(ANPP_{mod}\right)}_{t}\right]}^{2}}{n}},$$

where *R* ^2^ is the coefficient of determination between field‐observed ANPP data (*ANPP*_*obs*_) and modeled ANPP data (*ANPP*_*mod*_), which denotes a similar pattern between *ANPP*_*obs*_ and *ANPP*_*mod*_ and the fraction of *ANPP*_*obs*_ variation that can be explained by the model. *RMSE* is the root‐mean‐square error between *ANPP*_*obs*_ and *ANPP*_*mod*_, which represents biases that cause modeled ANPP data to differ from field‐observed ANPP data. n is the number of field ANPP observations included in the dataset for model validation.

As stated above, the model with the highest *R* ^2^ and lowest *RMSE* of the 52 regression models developed was identified as the best model for estimating the ANPP of the entire EASR (Taylor, 2001). When no model satisfies both maximum *R* ^2^ and minimum *RMSE*, the existing 52 models need to be optimized (Taylor, 2001). In this study, the optimal model was obtained by generating the averaged result of ANPP estimations using the model with the highest *R* ^2^ and the model with the lowest *RMSE* at the pixel scale.

The optimal ANPP estimation model (the Integrated ANPP_NDVI_ model) specific to the entire EASR in the Subregions Integrated Scheme was also determined through two steps: (1) optimal ANPP estimation models for three subregions in the EASR were obtained by developing model approaches that reflect the Entirety Overall Scheme, and (2) the best ANPP estimation models of the three subregions were integrated to generate the optimal model for the entire EASR. Finally, after comparing the Overall ANPP_NDVI_ model with the Integrated ANPP_NDVI_ model, the one presenting greater validation accuracy was used to estimate the ANPP of the entire EASR.

#### 2.3.2 Analysis of temporal dynamics of TANPP

TANPP time series trends for 1982–2013 were analyzed using a simple regression model. The turning points (TPs) at which trends in the TANPP time series significantly changed were identified using a piecewise linear regression (PLR) model (Toms & Lesperance, 2003) (Equation (4)).

(4) $$TANPP=\left\{\begin{array}{cc} \beta_{0}+\beta_{1}+\xi , & t\leq \alpha \\ \beta_{0}+\beta_{1}t+\beta_{2}\left(t+\alpha \right)+\xi & t>\alpha \end{array}\right\},$$

where *t* is the year from 1982 to 2013 and *TANPP* is the regional total aboveground net primary productivity per year. α is the estimated TP of the time series, which denotes the timing of a trend change. β_0_, β_1_, and β_2_ are regression coefficients, and ξ is the residual of the fit. β_1_ and β_1+_ β_2_ denote linear TANPP trends before and after the turning point, respectively. The three regression coefficients were determined through least‐square linear regression. In addition, a *t*‐test was conducted to evaluate the necessity of introducing a TP based on the following null hypothesis: “β_2_ is not significantly different from zero.” A *p* value of <.05 was considered significant. The TANPP trends for each subperiod defined by the TP were also analyzed.

## 3 RESULTS

### 3.1 Development and validation of the ANPP estimation model

Model accuracy levels directly affect the reliability of research conclusions. Accuracy assessments based on the remaining 25% of field‐observed ANPP data (Appendices S5 and S6) indicated that models with both maximum *R* ^2^ and minimum *RMSE* were absent from the 52 regression models fitted in the Entirety Overall Scheme or in the Subregions Integrated Scheme. According to the approaches for developing an ANPP estimation model, the Overall ANPP_NDVI_ model and the Integrated ANPP_NDVI_ model were composed of the fitted regressions meeting the maximum *R* ^2^ criteria and satisfying the minimum *RMSE* criteria among the 52 regression models in both the Entirety Overall Scheme and the Subregions Integrated Scheme, respectively.

**Figure 2:**
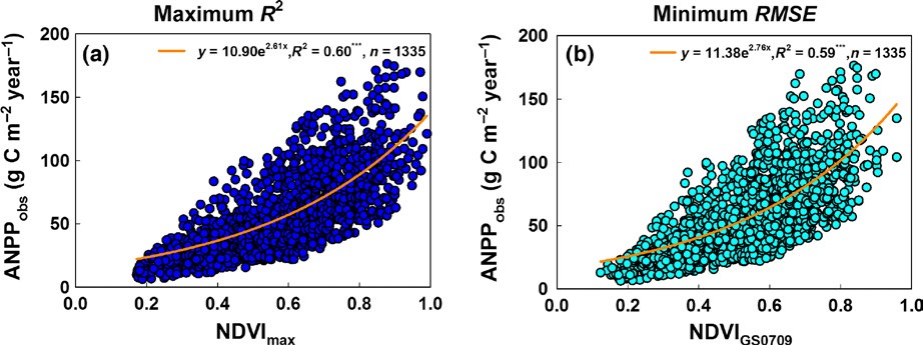
The relationship meeting the maximum *R* ^2^ criteria (a) and minimum *RMSE* (b) criteria between field‐observed ANPP data and the NDVI values of the corresponding composite period in the Entirety Overall Scheme. *R* ^2^ and *RMSE* denote the coefficient of determination and the root mean error, respectively. ANPP denotes the aboveground net primary productivity per year and per square meter. ANPP_obs_ denotes field‐observed ANPP data. *NDVI*_max_ denotes the annual maximum NDVI. *NDVI*_GS0709_ denotes the averaged NDVI of the period running from July to September. *** indicates that a regression equation was significant at the .001 level

In the Entirety Overall Scheme, the regression relationship between field‐observed ANPP and the annual maximum NDVI met the maximum *R* ^2^ criteria (Figure 2a). In addition, the regression correlation of field‐observed ANPP with the average NDVI of the period running from July to September satisfied the minimum *RMSE* criteria (Figure 2b). Fitted regressions with the maximum *R* ^2^ criteria (Figure 2a) and the minimum *RMSE* criteria (Figure 2b) between field‐observed ANPP and NDVI values together formed the Overall ANPP_NDVI_ model (Equation (5)). The validation of the Overall ANPP_NDVI_ model (Figure 4a) shows that the *R* ^2^and *RMSE* between the estimated ANPP and field‐observed ANPP were 0.58 and 17.24 g C m^−2^ yr^−1^, respectively, for the entire EASR (Figure 4a).

(5) $$ANPP\left(x,t\right)\;=\;\frac{1}{2}\left(10.90e^{2.61NDVI_{max}}+11.38e^{2.76NDVI_{GS0709}}\right),$$

where *t* is the year from 1982 to 2013 and *x* denotes the geographical position. *ANPP(x,t)* denotes the aboveground net primary productivity at position *x* in year *t*. *NDVI*_*max*_(*t*) denotes the annual maximum NDVI of year *t*, and *NDVI*_GS0709_(*t*) denotes the averaged NDVI of the period running from July to September in year *t*.

**Figure 3:**
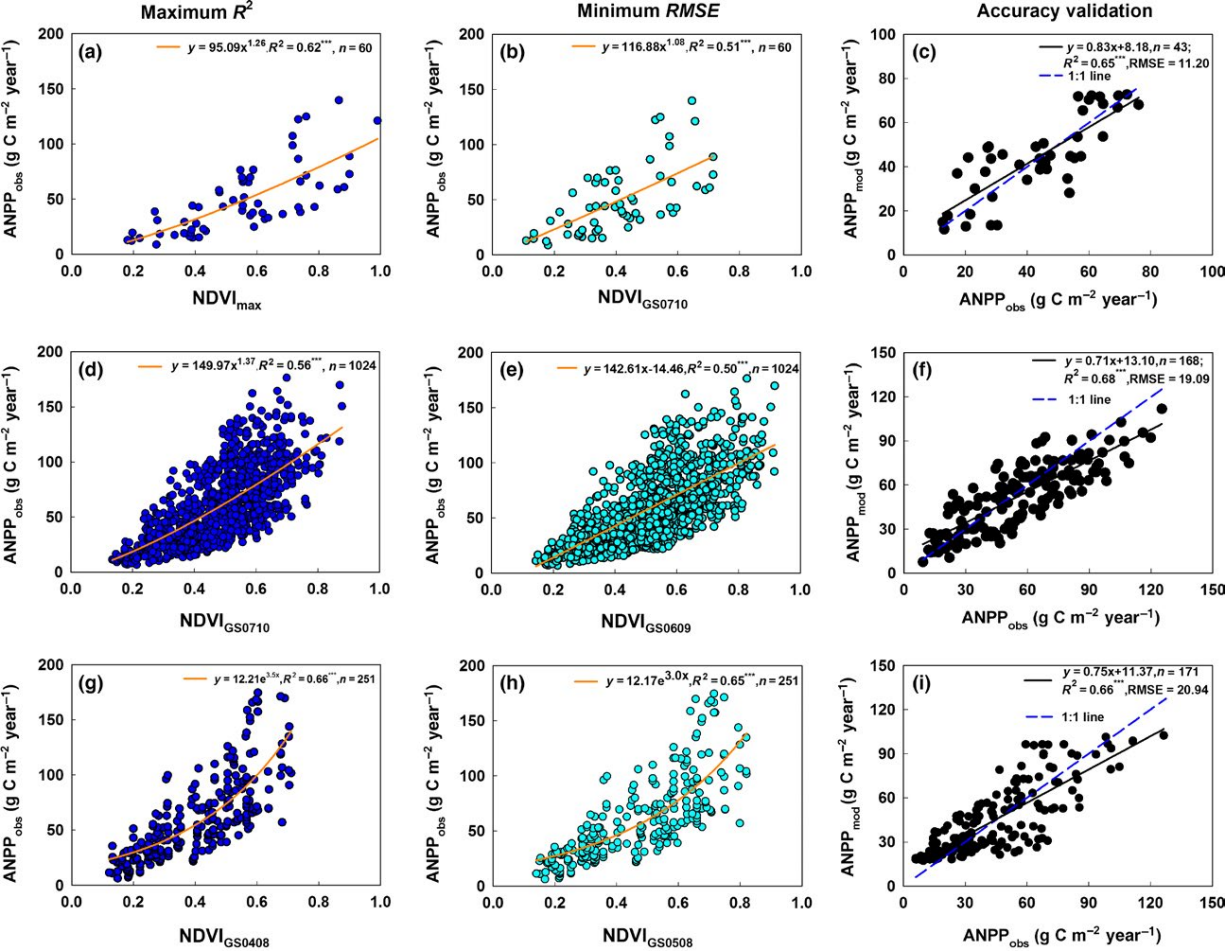
Fitted regressions that meet the maximum *R* ^2^ criteria and minimum *RMSE* criteria between field‐observed ANPP data and NDVI values of the corresponding composite period for the BKSSR (a, b), MPSSR (d, e), and TPSSR(g, h) in the Subregions Integrated Scheme, and the validation of the Integrated ANPP_NDVI_ model in the BKSSR (c), MPSSR (f), and TPSSR (i). *R* ^2^ and *RMSE* denote the coefficient of determination and the root mean error, respectively. ANPP denotes the aboveground net primary productivity per year and per square meter. ANPP_obs_ denotes field‐observed ANPP data. ANPP_mod_ denotes the modeled ANPP of the Integrated ANPP_NDVI_ model. *NDVI*_max_ denotes the annual maximum NDVI. *NDVI*_GS0710_, *NDVI*_GS0609_, *NDVI*_GS0408_, and *NDVI*_GS0508_ denote the averaged NDVI values for July to October, June to September, April to August, and May to August, respectively. *^**^ indicates that a regression equation was significant at the .001 level

In the Subregions Integrated Scheme, the fitted relationship between field‐observed ANPP and the annual maximum NDVI met the maximum *R* ^2^ criteria (Figure 3a) and that between field‐observed ANPP and the averaged NDVI of the period running from July to October satisfied the minimum *RMSE* criteria (Figure 3b) for the BKSSR. In addition, regression correlations of the field‐observed ANPP with the averaged NDVI of the period from July to October (Figure 3d) and the averaged NDVI of the period running from June to September (Figure 3e) satisfied the maximum *R* ^2^ and minimum *RMSE* criteria for the MPSSR. Regressions between field‐observed ANPP and the averaged NDVI for the period from April to August (Figure 3g) and between field‐observed ANPP and the averaged NDVI for the period from May to August (Figure 3h) separately met the maximum *R* ^2^ and minimum *RMSE* criteria for the TPSSR.

**Figure 4:**
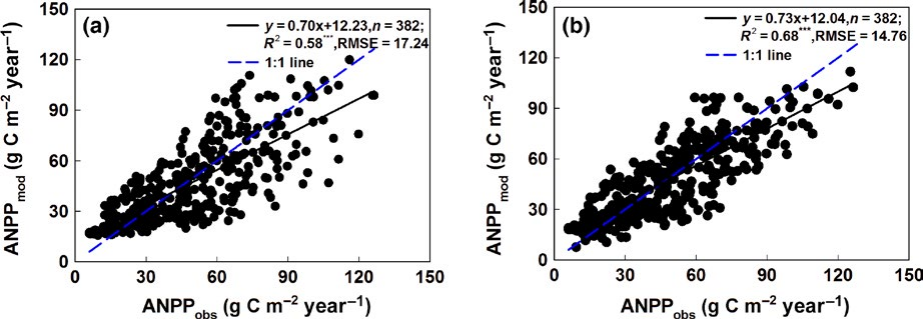
Validation of the Overall ANPP_NDVI_ model (a) and Integrated ANPP_NDVI_ model (b) for the entire Eurasian steppe region. ANPP denotes the aboveground net primary productivity per year and per square meter. ANPP_obs_ denotes field‐observed ANPP data. ANPP_mod_ denotes modeled ANPP data. *^**^ indicates that a regression equation was significant at the .001 level

In each subregion, the fitted regressions meeting the maximum *R* ^2^ and minimum *RMSE* criteria formed the best ANPP estimation model for the subregion. The best ANPP estimation models for the three subregions integrated together formed the Integrated ANPP_NDVI_ model (Equation (6)), which was optimal for ANPP estimations of the entire EASR. The validation of the Integrated ANPP_NDVI_ model showed that the *R* ^2^ and *RMSE* between the estimated ANPP and field‐observed ANPP were 0.65 and 11.20 g C m^−2^ yr^−1^ for the BKSSR (Figure 3c), 0.68 and 19.09 g C m^−2^ yr^−1^ for the MPSSR (Figure 3f), 0.66 and 20.94 g C m^−2^ yr^−1^ for the TPSSR (Figure 3i), and 0.68 and 14.76 g C m^−2^ yr^−1^ for the entire EASR (Figure 4b), respectively.

(6) $$ANPP\left(x,t\right)=\left\{\begin{array}{cc} \frac{1}{2}\left(95.09NDVI_{max}{\left(t\right)}^{1.26}+116.88NDVI_{GS0710}{\left(t\right)}^{1.08}\right), & BKSSR\\ \frac{1}{2}\left(149.97NDVI_{GS0710}{\left(t\right)}^{1.37}+142.61NDVI_{GS0609}\left(t\right)-14.46\right), & MPSSR\\ \frac{1}{2}\left(\right.12.21e^{3.48NDVI_{GS0408}\left(t\right)}+12.17e^{3.01NDVI_{GS0508}\left(t\right)}, & TPSSR \end{array}\right.$$

where *t* is the year from 1982 to 2013 and *x* denotes the geographic position. *ANPP (x,t)* denotes the aboveground net primary productivity at position *x* in year *t*. *NDVI*_max_ (*t*) denotes the annual maximum NDVI for year *t*. *NDVI*_GS0710_ (*t*), *NDVI*_GS0609_ (*t*), *NDVI*_GS0408_ (*t*), and *NDVI*_GS0508_ (*t*) denote, respectively, the averaged NDVI values for July to October, June to September, April to August, and May to August for year *t*.

Comparisons between accuracy assessments of the Overall ANPP_NDVI_ model and the Integrated ANPP_NDVI_ model (Figure 4) showed that the NDVI‐based ANPP estimation models from both the Entirety Overall Scheme and the Subregions Integrated Scheme effectively simulated ANPP variations for the entire EASR. However, the Integrated ANPP_NDVI_ model performed better than the Overall ANPP_NDVI_ model. For the entire EASR, the *R* ^2^ between modeled ANPP and field‐observed ANPP increased from 0.58 in the Overall ANPP_NDVI_ model to 0.68 in the Integrated ANPP_NDVI_ model, and the *RMSE* decreased from 17.24 g C m^−2 ^yr^−1^ to 14.76 g C m^−2^ yr^−1^. Therefore, the Integrated ANPP_NDVI_ model was used to simulate ANPP variations for the entire EASR in this study.

### 3.2 Geographic ANPP patterns

The Integrated ANPP_NDVI_ model developed via the Subregions Integrated Scheme (Equation 6) was applied to estimate the ANPP value for 1982–2013 for the entire EASR. The mean values of annual ANPP for the entire EASR and its three subregions were evaluated using zonal statistics (Table 1). The mean annual ANPP of the entire EASR was recorded as 43.78 ± 22.77 g C m^−2^ yr^−1^. For the three subregions, the BKSSR had the lowest mean annual ANPP with a value of 37.70 ± 16.60 g C m^−2^ yr^−1^. In addition, the MPSSR had the highest mean annual ANPP with a value of 52.86 ± 24.78 g C m^−2^ yr^−1^. The mean annual ANPP of the TPSSR fell between those of the BKSSR and MPSSR at a value of 46.98 ± 28.94 g C m^−2^ yr^−1^.

**Table 1:** Mean (ANPP) and total values (TANPP) of annual ANPP in the Eurasian steppe region and three subregions. ANPP denotes the aboveground net primary productivity per year and per square meter. TANPP denotes the regional total aboveground net primary productivity per year. EASR denotes the Eurasian steppe region, BKSSR denotes the Black Sea–Kazakhstan steppe subregion, MPSSR denotes the Mongolian Plateau steppe subregion, and TPSSR denotes the Tibetan Plateau alpine steppe subregion. Mean denotes the mean value of annual ANPP for a region. *SD* denotes the standard deviation of mean annual ANPP

	Area (10^4^ × km^2^)	ANPP (Mean ±* SD*, g C m^−2^ yr^−1^)	TANPP (Tg C yr^−1^)
BKSSR	458.85	37.70 ± 16.60	173.08
MPSSR	252.18	52.86 ± 24.78	133.31
TPSSR	154.48	46.98 ± 28.94	72.58
EASR	865.51	43.78 ± 22.77	378.97

Total annual ANPP (TANPP) values for the entire EASR and its three subregions were generated by combining the mean ANPP with the region area (Table 1). For 1982 to 2013, the multiyear average TANPP was recorded as 378.97 Tg C yr^−1^ for the entire EASR. TANPP values were the highest in the BKSSR at 173.08Tg C yr^−1^ and the lowest in the TPSSR at 72.58 Tg C yr^−1^. Moreover, the TANPP of the MPSSR was recorded as 133.31 Tg C yr^−1^.

**Figure 5:**
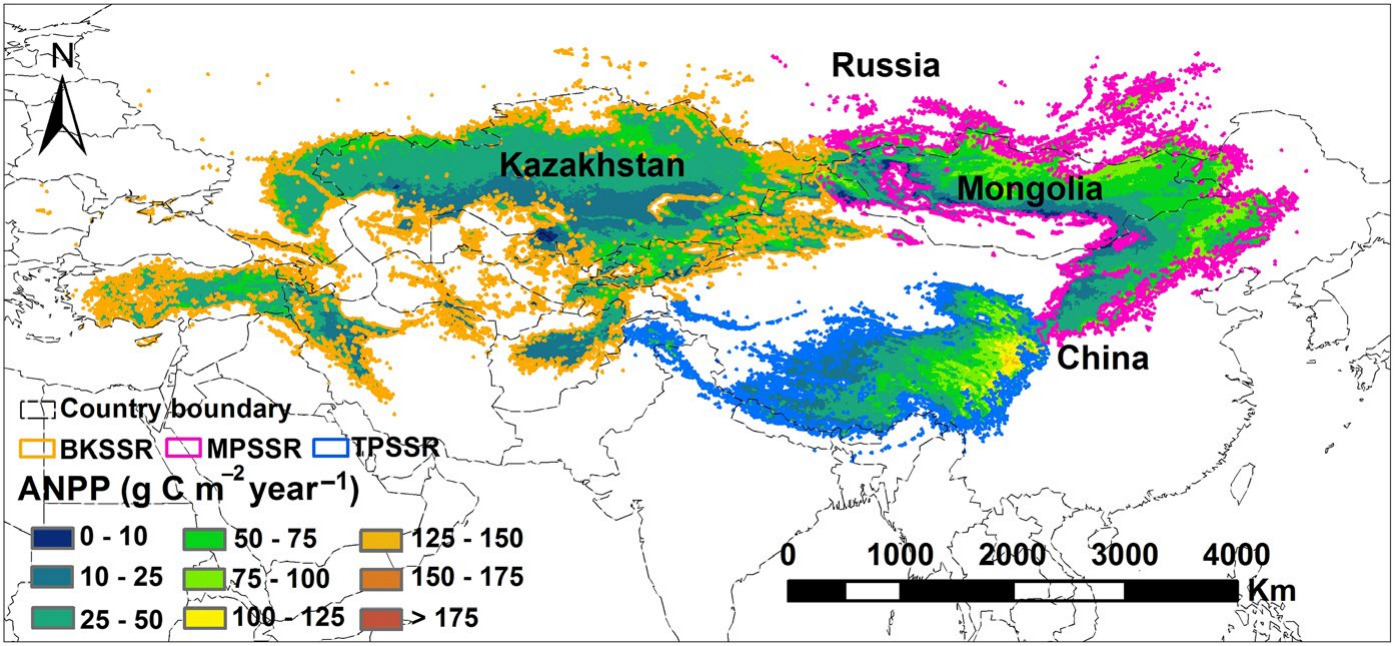
The spatial pattern of the multiyear (1982–2013) averaged ANPP for the Eurasian steppe region. ANPP denotes the aboveground net primary productivity per year and per square meter. BKSSR denotes the Black Sea–Kazakhstan steppe subregion, MPSSR denotes the Mongolian Plateau steppe subregion, and TPSSR denotes the Tibetan Plateau alpine steppe subregion

The multiyear average ANPP exhibited pronounced spatial variations in the EASR (Figure 5) which corresponded to different grassland types reflecting variations in hydrothermal conditions (Appendices S3 and S7). Regarding climatic conditions, the BKSSR and the MPSSR are located in temperate semi‐arid regions in which water levels typically limit vegetation growth (Appendices S1 and S2). In the BKSSR and MPSSR, desert steppes, typical steppes, meadow steppes, and meadows were found along an increasing precipitation gradient from the center to the boundaries (Appendices S1 and S3), and the increasing ANPP values reached 27.69 g C m^−2^ yr^−1^, 47.61 g C m^−2^ yr^−1^, 56.30 g C m^−2^ yr^−1^, and 75.74 g C m^−2^ yr^−1^ (Appendix S7). In addition, the TPSSR was subjected to a unique cold alpine climate. From the northwest to the southeast of the TPSSR with increasing precipitation and temperatures, representative vegetation types included alpine steppes and alpine meadows (Appendices S1 and S3) where ANPP values reached 51.11 g C m^−2^ yr^−1^ and 33.93 g C m^−2^ yr^−1^, respectively (Appendix S7).

### 3.3 Temporal TANPP dynamics

**Figure 6:**
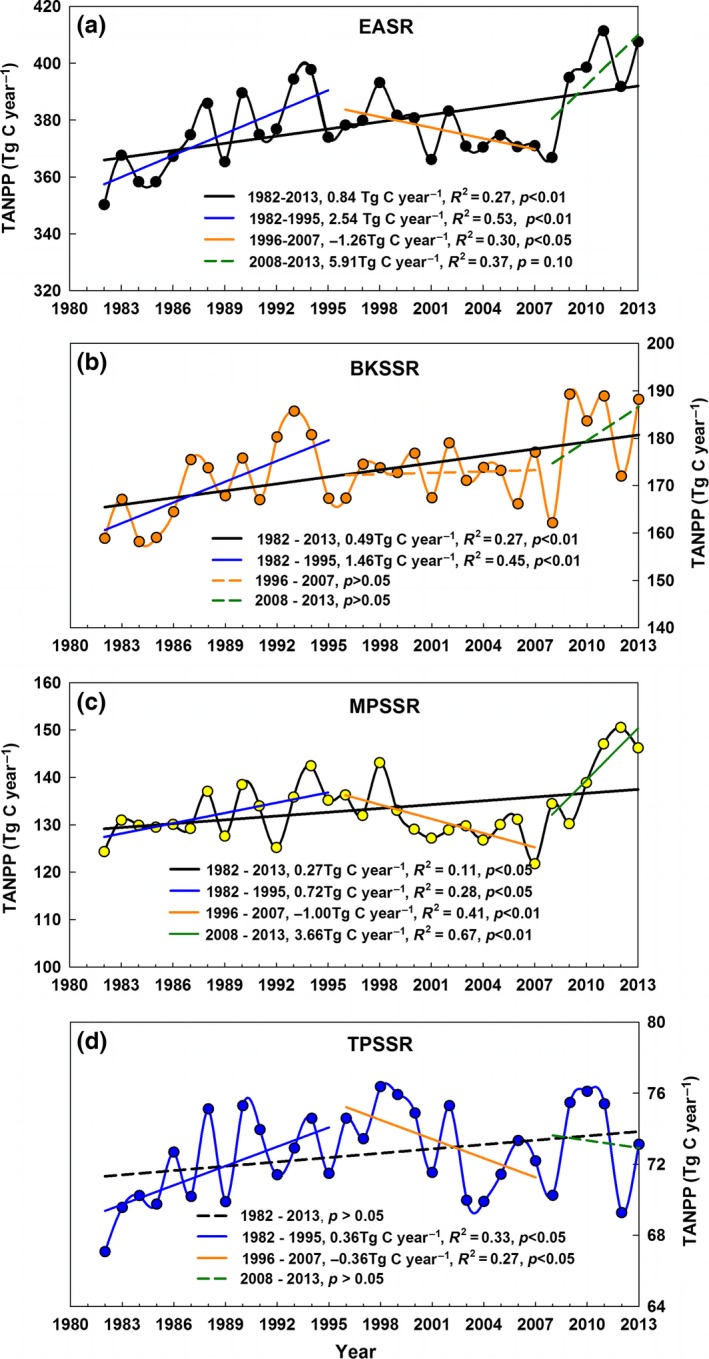
Interannual variations in the TANPP time series for the Eurasian steppe region from 1982 to 2013. Three subperiods with significantly different TANPP trends were identified through piecewise linear regression. TANPP denotes the regional total aboveground net primary productivity level per year. EASR (a) denotes the Eurasian steppe region, BKSSR (b) denotes the Black Sea–Kazakhstan steppe subregion, MPSSR (c) denotes the Mongolian Plateau steppe subregion, and TPSSR (d) denotes the Tibetan Plateau alpine steppe subregion

Interannual variations in TANPP for 1982–2013 were analyzed using a simple regression model (Figure 6). For the entire EASR, TANPP values significantly increased from 1982 to 2013 at an annual rate of 0.84 Tg C yr^−1^ or 0.49% (Figure 6a). TANPP changes in the BKSSR and the MPSSR were similar to those for the entire EASR and exhibited an obvious increase (Figure 6b, c). However, the TANPP of the TPSSR showed no significant changes (Figure 6d).

EASR's TANPP variations were not continuous over the 32‐year period. The piecewise linear regression between EASR's TANPP and time (year) indicated that 1995 and 2007 were two turning points at which the EASR's TANPP time series significantly changed (Figure 6a). The EASR's TANPP experienced a significant increase from 1982 to 1995, followed by a marked decrease that occurred from 1996 to 2007. After 2008, the EASR's TANPP increased slightly (*p *=* *0.10).

Changes in the EASR's TANPP over the three subperiods constituted a superposition of variation trends in TANPP within the three subregions (Figure 6b, c, d). From 1982 to 1995, TANPP values in the BKSSR, MPSSR, and TPSSR increased markedly. From 1996 to 2007, TANPP values in the MPSSR and TPSSR significantly decreased, while TANPP in the BKSSR showed no significant change. From 2008 to 2013, TANPP in the MPSSR significantly increased, while that in BKSSR and TPSSR did not significantly change.

## 4 DISCUSSION

### 4.1 ANPP estimation model development theories

The NDVI could be used to model the spatiotemporal dynamics of ANPP for the entire EASR. Additionally, the best composite period of monthly NDVI data for estimating annual ANPP varied according to regional climatic conditions and vegetation types. The optimal composite period of monthly NDVI data for annual ANPP estimations included the middle to late growing season (June to September or July to October) in the MPSSR, the annual maximum NDVI (NDVI_max_) and the middle–late growing season (July to October) in the BKSSR, and the early–middle growing season (April to August or May to August) in the TPSSR.

For the MPSSR, climatic patterns are semi‐arid (Table 2), according to the categories of climatic conditions presented in Quan, Han, Utescher, Zhang, and Liu (2013) based on the climate index (*CI*_*Köppen*_) proposed by Köppen (1923) (Appendix S8). Therefore, water is a major factor limiting vegetation growth in this subregion. Therefore, the phenological period is affected not only by temperature but also by precipitation in this subregion. In addition, biomass normally peaks later in the growing season (Barnes, Tieszen, & Ode, 1983; Reed et al., 1994). Therefore, the averaged NDVI from the middle–late growing season can be used to effectively assess annual ANPP variations in the MPSSR.

**Table 2:** The optimal composite period of monthly NDVI data for annual ANPP estimations of different regions. BKSSR denotes the Black Sea–Kazakhstan steppe subregion, MPSSR denotes the Mongolian Plateau steppe subregion, and TPSSR denotes the Tibetan Plateau alpine steppe subregion. *CI*_*Köppen*_= MAP/(MAT+33), where *MAP* denotes the mean annual precipitation, and *MAT* denotes the mean annul temperature

	*CI*_*köppen*_	Climate regime	The optimal NDVI‐based variable
MPSSR	9.22	Semi‐arid (5.70–13.60)	Middle–late growing season averaged NDVI
BKSSR	7.95		Annual maximum NDVI
Desert steppes in the BKSSR	6.50		
TPSSR	15.31	Semi‐humid (13.60–15.60)	Early–middle growing season averaged NDVI

For the BKSSR, the climate, similar to that of the MPSSR, is semi‐arid (Table 2). Therefore, the averaged NDVI of the middle–late growing season is a key variable for assessing annual ANPP variations. However, according to the climate index (*CI*_*Köppen*_), vegetation in the BKSSR is subjected to more severe drought stress compared to the MPSSR. More specifically, desert steppes account for over 50% of the BKSSR area (Appendix S3). Desert steppes follow less predictable phenological patterns (Barnes et al., 1983; Reed et al., 1994) because they depend on less reliable precipitation events (Rauzi & Dobrenz, 1970). For desert steppes, the annual maximum NDVI can effectively reflect annual productivity levels. Therefore, ANPP variations of the entire BKSSR should be assessed by combing the annual maximum NDVI with the averaged NDVI for the middle–late growing season.

For the TPSSR, climatic patterns in the TPSSR are semi‐humid as a whole (Table 2). Therefore, the phenological period in the TPSSR is relatively stable owing to weak precipitation limitations affecting vegetation growth. Moreover, biomass typically reaches its maximum value at the beginning or middle of the summer season (Barnes et al., 1983; Reed et al., 1994). As a result, the averaged NDVI of the early–middle growing season reflects annual ANPP variations in the TPSSR.

In this study, the NDVI was a good indicator of ANPP for the EASR, which further confirmed conclusions from previous studies (An et al., 2013; Goward & Dye, 1987; Irisarri et al., 2012; Paruelo et al., 1997; Tucker, 1979; Tucker, Vanpraet, Sharman, & Van Ittersum, 1985). However, in the ANPP estimation models, most previous studies used NDVI values from a predefined time period based on the “normal,” or mean, growing season or a subjective time period such as calendar months (i.e., from April to October, from May to September, etc.) (Gu et al., 2013; Guo et al., 2012; Paruelo et al., 1997). This method may have affected the robustness of models (Reed et al., 1996). Only a few studies conducted in the arid rangelands located in Senegal (Fuller, 1998) and central Australia (Hobbs, 1995) explored the important composite periods of the NDVI in ANPP estimation models for the specific regions.

This study suggested that the averaged NDVI of the middle–late growing season or maximum NDVI was strongly related to ANPP in the MPSSR and the BKSSR, in which the climate was semi‐arid. This result agreed with conclusions obtained from research conducted in arid regions located in Africa (Fuller, 1998; Rasmussen, 1992) and arid rangeland located in central Australia (Hobbs, 1995). However, studies of composite periods of the NDVI in ANPP estimation models of alpine ecosystems, such as the Tibetan Plateau alpine steppes, have not yet been reported. This study enriched knowledge of relationships between the NDVI and ANPP in alpine ecosystems.

In conclusion, composite periods of NDVI data should be selected according to the climatic conditions and vegetation types found in a given study area when NDVI data are applied in empirical annual ANPP estimation models. Our analysis results show that the early–middle growing season averaged NDVI, the middle–late growing season averaged NDVI, and the annual maximum NDVI should, respectively, be applied for semi‐humid regions, semi‐arid regions, and desert vegetation in semi‐arid regions.

### 4.2 Accuracy of the ANPP estimation model

The Integrated ANPP_NDVI_ model is reliable and can be used to estimate annual ANPP variations for the entire EASR. To assess the robustness of the ANPP estimation model for the entire EASR, ANPP values estimated from the Integrated ANPP_NDVI_ model were compared to values reported in previous studies. Because ANPP estimations specific to the entire EASR have not yet been reported, we compared the estimated annual ANPP values in this study with values reported in previous studies (Gao et al., 2013; Jiang et al., 2015; Ma, Liu, et al., 2008; Yang, Fang, Pan, & Ji, 2009) for different grassland types in Inner Mongolian temperate and Tibetan Plateau alpine grassland areas.

According to the simulated ANPP values obtained from the Integrated ANPP_NDVI_ model (Appendix S9), the mean annual ANPP values of desert steppes, typical steppes, and meadow steppes all fell within ranges reported in previous studies (Gao et al., 2013; Guo et al., 2012; Hu, Fan, Zhong, & Yu, 2007; Ma, Fang, Yang, & Mohammat, 2010; Ma, Liu et al., 2010; Yang, Fang, Ma, Guo, & Mohammat, 2010). Moreover, the mean annual ANPP values of alpine steppes and alpine meadows were comparable to values reported in previous studies (Jiang et al., 2015; Ma, Fang, Yang, & Mohammat, 2010; Ma, Liu et al., 2010). In addition, the spatial distributions of ANPP for the Tibetan Plateau alpine grasslands and the Inner Mongolia temperate grasslands were consistent with the conclusions of previous studies (Guo et al., 2012; Yang et al., 2009).

It should be noted that some uncertainties may exist in the ANPP estimation model. Similar to most traditional studies based on field investigation, our study design did not allow for quantitative assessment of the sampling quality. The field‐observed ANPP data in this study combined multiple field surveys and datasets from previous studies (a list of the data sources can be found in the Appendix S11) without a consistent sampling design. Moreover, the previous studies disproportionally focused on the Mongolian Plateau steppe subregion and the Tibetan Plateau steppe subregion, while there were only a few samples from the Black Sea‐Kazakhstan steppe subregion owing to either its remote location or political restrictions (Li et al., 2015). The bias of the spatial distribution of ANPP field sites might generate some uncertainties in the ANPP estimation model. Unfortunately, we are unable to evaluate the uncertainties generated from this spatial bias.

However, in our opinion, the spatial bias of ANPP field sites would have little effect on the estimation of ANPP. The theoretical connection between ANPP and the NDVI is the Monteith's (1981) equation (Paruelo et al., 1997). In addition, the NDVI is a linear indicator of absorbed photosynthetic active radiation (Sellers, 1985, 1987; Sellers, Berry, Collatz, Field, & Hall, 1992). Therefore, the equation was applicable for the entire EASR, which expressed the relationships between ANPP and the NDVI based on few field‐observed ANPP data of the BKSSR.

### 4.3 The importance of the EASR in global carbon cycling

The EASR has been playing a significant role in global carbon sequestration. EASR's TANPP was found to be 378.97 Tg C yr^−1^ (Table 1), which represented 8.18%–36.03% of that of all grasslands. According to previous studies (Bazilevich et al., 1971; Parton et al., 1995; Whittaker & Likens, 1975; Xia et al., 2014), the TANPP of all grasslands is 1423 Tg C yr^−1^ – 4635 Tg C yr^−1^ (Appendix S10). EASR's TANPP was higher compared to the TANPP for grasslands in North America, South America, and Africa (Appendix S10). The mean value of annual ANPP for the entire EASR was recorded as 43.78 ± 22.77 g C m^−2^ yr^−1^ (Table 1), which was lower than that for the global grasslands average (Bazilevich et al., 1971; Parton et al., 1995; Whittaker & Likens, 1975; Yang et al., 2008; Xia et al., 2014) and North American (Bazilevich et al., 1971; Xia et al., 2014), South American (Xia et al., 2014), and African grasslands (Xia et al., 2014) (Appendix S10).

For the three subregions studied, TANPP values were found to be the highest in the BKSSR because of its vast area (which accounted for 53.01% of the EASR's area), despite its low mean annual ANPP value. While the BKSSR has not been studied at length in the past, it plays an indispensable role in global carbon cycling. Different characteristics of ANPP among the three subregions showed that the mean annual ANPP of the BKSSR was lower compared to both the MPSSR and the TPSSR (Table 1). There were two main reasons for this difference. One was that vegetation growth in the BKSSR was under more severe drought stress compared to the MPSSR because the MAT in the BKSSR was significantly higher compared to the MPSSR despite the MAP being comparable between them (Appendix S1). The other reason was that ANPP was affected by not only annual precipitation but also the precipitation seasonal distribution (Guo et al., 2012). Rain and heat occurred over the same period both in the MPSSR and in the TPSSR (Appendix S2). However, the rainy season did not coincide with the hot season in the BKSSR (Appendix S2), which was not good for annual herbaceous growth. The mean annual ANPP of the TPSSR was lower that of the MPSSR owing to the limitations of low temperature for vegetation growth under the special cold alpine environment of the Tibetan Plateau (Fang et al., 2005; Kato et al., 2006).

### 4.4 TANPP variation trends

The TANPP for the entire EASR showed an obvious increase from 1982 to 2013. Compared to other important natural grassland regions, the increasing TANPP of EASR estimated in this study (0.53 Tg C yr^−1^, 1982–2006) was lower than that of the global grasslands average (2.43 Tg C yr^−1^) and African grasslands (1.21 Tg C yr^−1^) and higher than that of North (0.33 Tg C yr^−1^) and South (‐0.44 Tg C yr^−1^) American grasslands (Xia et al., 2014). Despite a statistically significant uptrend in EASR's TANPP for the past three decades (1982–2013), change trends were not temporally homogeneous over the whole period (Figure 6). The EASR's TANPP significantly increased from 1982 to 1995, followed by a marked decrease from 1996 to 2007 and a weakening uptrend from 2008 to 2013.

Vegetation growth was influenced by precipitation in the BKSSR and the MPSSR (Bao et al., 2014, 2015; Jiao et al., 2017), and by temperature in the TPSSR (Zhang et al., 2014). During 1982–1995, the TANPP of EASR increased significantly because TANPP increases in the three subregions occurred owing to increasing precipitation in the BKSSR and MPSSR (Bao et al., 2015) and warming in the TPSSR (Piao et al., 2011; Zhou et al., 2001). In the period of 1996–2007, EASR's TANPP decreased apparently because of a decrease in TANPP in the MPSSR and TPSSR, which was caused by decreasing precipitation, especially summer precipitation in the MPSSR (Bao et al., 2014, 2015; Piao et al., 2011) and by decreasing temperature in the TPSSR (Piao et al., 2011). During 2008–2013, EASR's TANPP increased weakly owing to increasing summer precipitation in the MPSSR (Bao et al., 2015).

The fact that the TANPP trend in EASR reversed from positive during 1982–1995 to negative during 1996–2007 was similar to TANPP variations in other grasslands (e.g., North America, South America and Africa). Xia et al. (2014) showed that the increase in the TANPP of other grasslands globally during the period of 1982–2006 reversed around 1994. In addition, a similar prominent reversal occurred in vegetation NDVI trends by the mid or late‐1990s for the temperate ecosystems of Eurasia, with a pronounced increase occurring before the mid or late 1990s and a decline (or a weakened increase) occurring afterward (Mohammat et al., 2013; Piao et al., 2011). This conclusion was further confirmed by our analysis results. In addition, our results based on an extended period (1980s to the early 2010s) from previous studies (1980s to mid‐2000s) also showed that 2007 constituted another turning point at which variations in the EASR's TANPP clearly changed. The EASR's TANPP showed a marked decrease before 2007 and increased slightly after 2007. This result furthered our understanding of changes in vegetation growth in the temperate ecosystems of Eurasia.

It is important to note that the interpretations of the causes of variation trends in EASR's TANPP mentioned above were generated by reviewing current studies conducted in the MPSSR, TPSSR, and Kazakhstan. The scientific validity of these interpretations needs to be further confirmed in the future. The mechanisms of the temporal dynamics of TANPP are complex in the EASR because of its vast area, complex topography, and diverse climate regimes, which will be discussed in greater detail and depth in our future studies.

## 5 CONCLUSIONS

To the best of our knowledge, this study was the first to assess the role of the entire EASR in the global carbon cycle. According to our analysis, although EASR's ANPP is lower than that of North American, South American, and African grasslands, EASR's TANPP is higher than that of grasslands in North America, South America, and Africa, accounting for 8.18%–36.03% of that of all grasslands. The EASR's TANPP displayed an obvious uptrend over the past three decades, for which the increasing rate was higher than that in North and South American grasslands over the same period. This result indicates the indispensable and ever‐increasing role of the EASR in global carbon sequestration. Moreover, there were several important turning points of the EASR's TANPP trend in the past 30 years.

In addition, our analysis also demonstrates that the best composite period of NDVI data for annual ANPP estimation varies with climate and vegetation in the study region. More specifically, the early–middle growing season averaged NDVI, the middle–late growing season averaged NDVI, and the annual maximum NDVI should be, respectively, applied to semi‐humid regions, semi‐arid regions, and desert vegetation in semi‐arid regions.

## CONFLICT OF INTEREST

None declared.

## DATA ACCESSIBILITY

Field‐observed ANPP data from published papers in this study can be available by contacting with Guirui Yu (yugr@igsnrr.ac.cn) and Cuicui Jiao (jiaocuicui1987@sina.cn).
