# Supplementary material for: Analysis of spatial and temporal patterns of aboveground net primary productivity in the Eurasian steppe region from 1982 to 2013
Source: Ecol Evol. 2017 Jun 6;7(14):5149–62. doi: 10.1002/ece3.3027 (PMC5528232; doi:10.1002/ece3.3027)
Supplement: Supplementary file 1 [file ECE3-7-5149-s001.docx]

# Supporting Information

**Appendix S1** Spatial patterns of the MAP (a) and MAT (b) in the Eurasian steppe region. MAP denotes the mean annual precipitation, and MAT denotes the mean annual temperature.


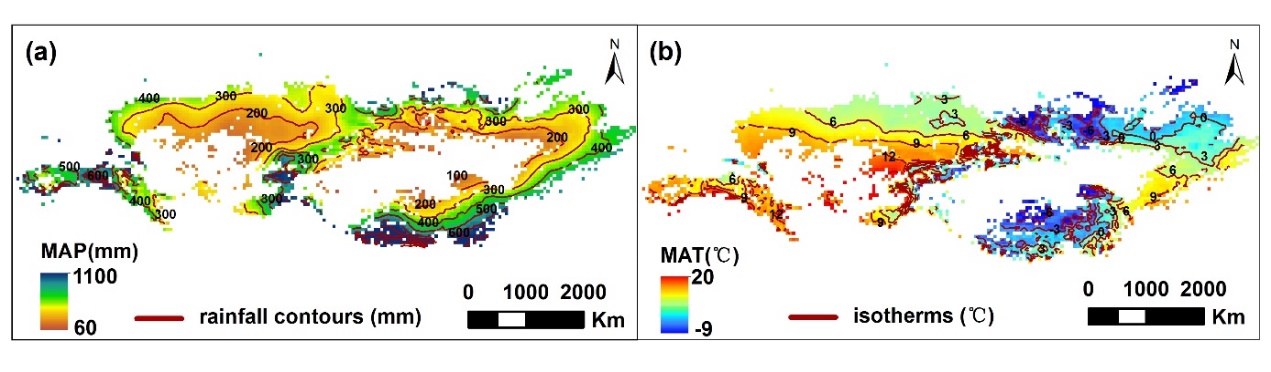


**Appendix S2** Seasonal variations of precipitation and temperature in the Eurasian steppe region. EASR (a, e) denotes the Eurasian steppe region, BKSSR (b, f) denotes the Black Sea–Kazakhstan steppe sub-region, MPSSR (c, g) denotes the Mongolian Plateau steppe sub-region, and TPSSR (d, h) denotes the Tibetan Plateau alpine steppe sub-region.


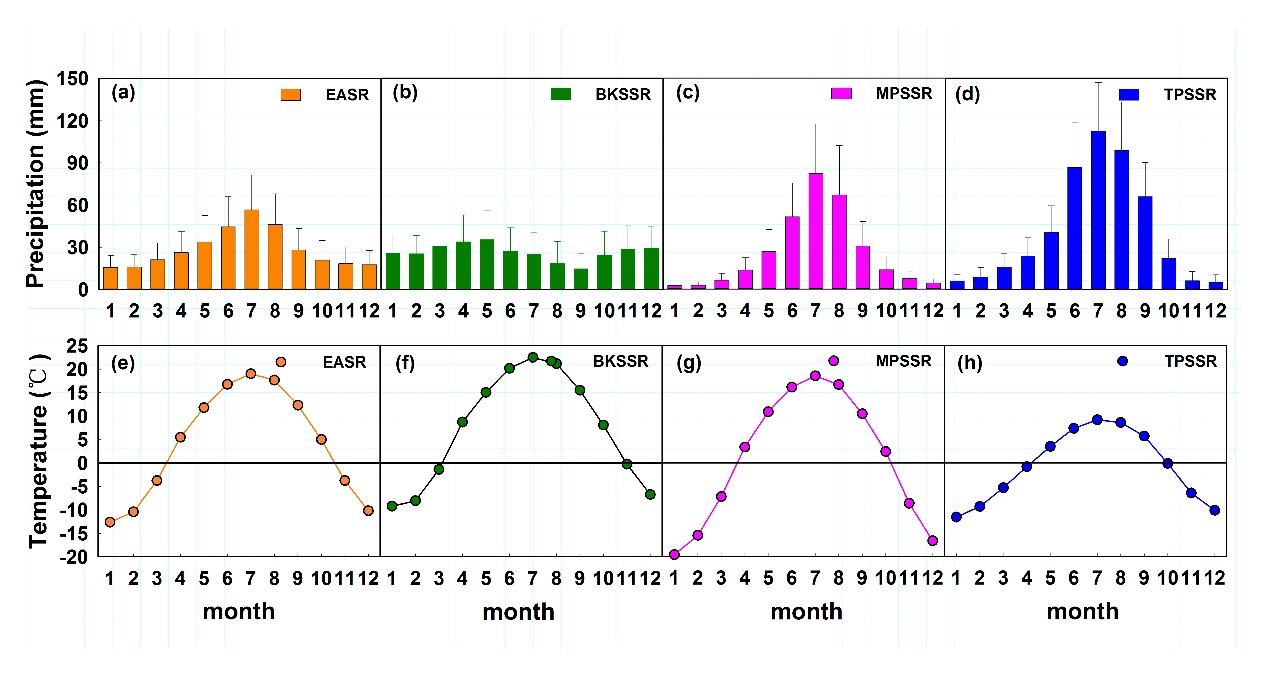


**Appendix S3** The spatial distribution of grassland vegetation along precipitation gradients in the Eurasian steppe region^[[1]](#footnote-1)^.


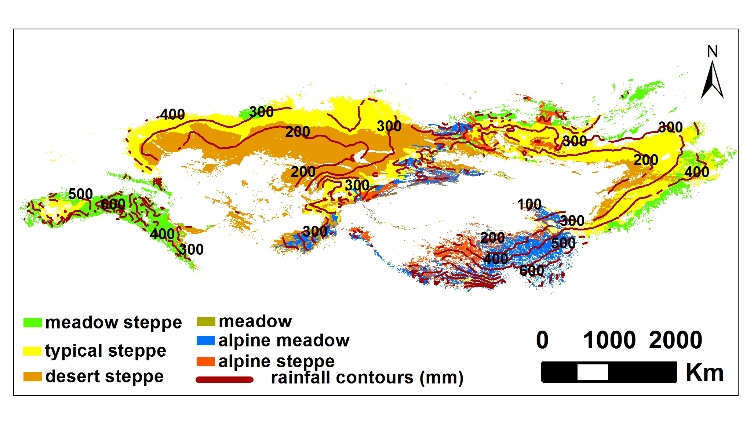


**Appendix S4** The number of site-year ANPP observations collected during various sub-periods running from 1982 to 2013.ANPP_obs_ denotes field-observed net primary productivity data. Numbers above bars denote the number of site-year ANPP observations collected during each sub-period.

**
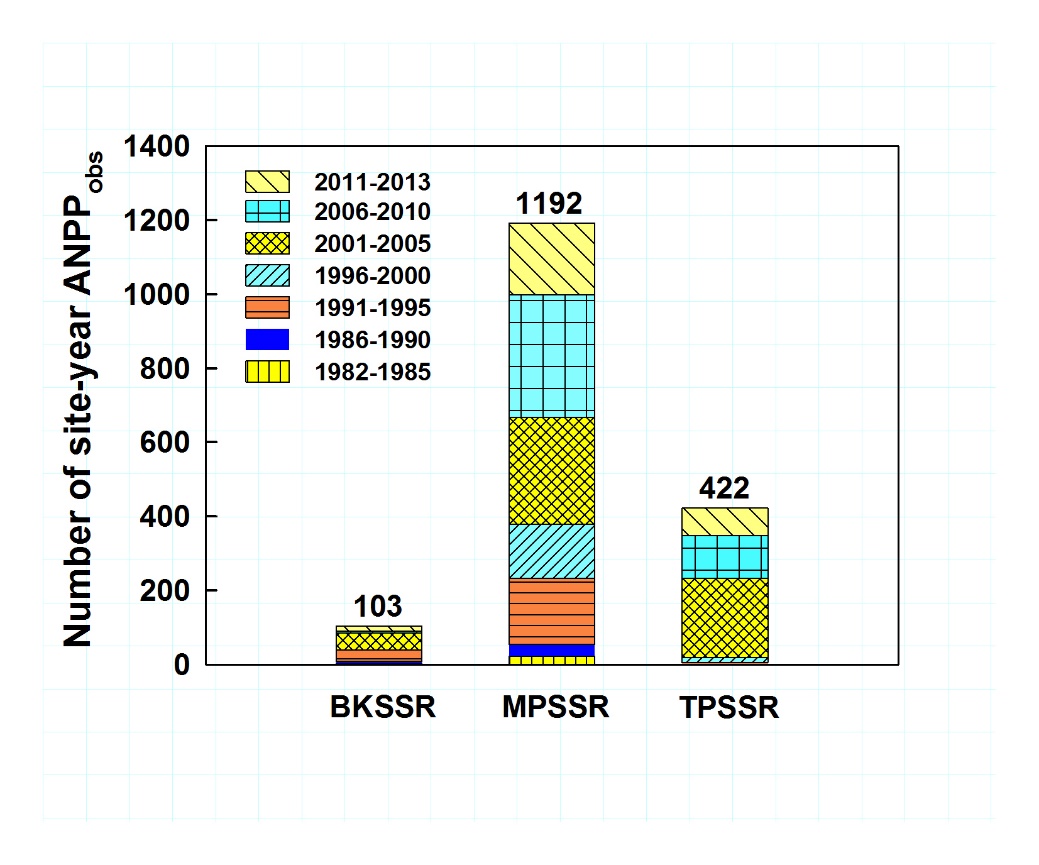
**

**Appendix S5** Accuracy assessments based on the remaining 25% of field-observed ANPP data of the 52 regression models developed in the Entirety Overall Scheme.$R^{2}$ and $RMSE$ denote the coefficient of determination and the root mean error, respectively. NDVI*_max_* represents the annual maximum NDVI. NDVI*_GS0410_*, NDVI*_GS0510_*, NDVI*_GS0610_*, NDVI*_GS0710_*, NDVI*_GS0409_*, NDVI_GS_*_0509_*, NDVI_GS_*_0609_*, NDVI_GS_*_0408_*, NDVI_GS_*_0508_*, NDVI*_GS0608_*, NDVI*_GS0708_*, and NDVI*_GS0709_* respectively denote the averaged NDVI of period from April to October, from May to October, from June to October, from July to October, from April to September, from May to September, from June to September, from April to August, from May to August, from June to August, from July to August, and from July to September.

|  | | **Forms of regression models** | 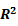 | 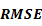 |
| --- | --- | --- | --- | --- |
| **Maximum annual NDVI** | NDVI*_max_* | liner regression | 0.530 | 20.323 |
|  |  | power exponent function | 0.620 | 20.493 |
|  |  | exponential function | 0.630 | 22.899 |
|  |  | logarithmic function | 0.490 | 22.670 |
| **Averaged growing season NDVI** | NDVI*_GS0410_* | liner regression | 0.450 | 20.631 |
|  |  | power exponent function | 0.540 | 21.415 |
|  |  | exponential function | 0.510 | 21.857 |
|  |  | logarithmic function | 0.430 | 22.838 |
|  | NDVI*_GS0510_* | liner regression | 0.490 | 20.481 |
|  |  | power exponent function | 0.570 | 21.002 |
|  |  | exponential function | 0.550 | 21.074 |
|  |  | logarithmic function | 0.460 | 22.850 |
|  | NDVI*_GS0610_* | liner regression | 0.500 | 20.396 |
|  |  | power exponent function | 0.580 | 20.721 |
|  |  | exponential function | 0.560 | 21.848 |
|  |  | logarithmic function | 0.470 | 20.197 |
|  | NDVI*_GS0710_* | liner regression | 0.500 | 20.578 |
|  |  | power exponent function | 0.580 | 20.892 |
|  |  | exponential function | 0.570 | 20.541 |
|  |  | logarithmic function | 0.460 | 23.126 |
|  | NDVI*_GS0409_* | liner regression | 0.470 | 20.508 |
|  |  | power exponent function | 0.550 | 21.221 |
|  |  | exponential function | 0.530 | 21.388 |
|  |  | logarithmic function | 0.450 | 22.688 |
|  | NDVI*_GS0509_* | liner regression | 0.500 | 20.352 |
|  |  | power exponent function | 0.580 | 20.807 |
|  |  | exponential function | 0.560 | 20.652 |
|  |  | logarithmic function | 0.470 | 22.632 |
|  | NDVI*_GS0609_* | liner regression | 0.520 | 20.254 |
|  |  | power exponent function | 0.600 | 20.515 |
|  |  | exponential function | 0.580 | 20.218 |
|  |  | logarithmic function | 0.480 | 22.613 |
|  | NDVI*_GS0408_* | liner regression | 0.440 | 20.638 |
|  |  | power exponent function | 0.530 | 21.494 |
|  |  | exponential function | 0.500 | 21.835 |
|  |  | logarithmic function | 0.430 | 22.495 |
|  | NDVI*_GS0508_* | liner regression | 0.490 | 20.396 |
|  |  | power exponent function | 0.570 | 20.955 |
|  |  | exponential function | 0.550 | 20.941 |
|  |  | logarithmic function | 0.460 | 22.441 |
|  | NDVI*_GS0608_* | liner regression | 0.510 | 20.207 |
|  |  | power exponent function | 0.590 | 20.539 |
|  |  | exponential function | 0.570 | 20.334 |
|  |  | logarithmic function | 0.470 | 23.070 |
|  | NDVI*_GS0708_* | liner regression | 0.510 | 20.360 |
|  |  | power exponent function | 0.600 | 20.672 |
|  |  | exponential function | 0.580 | 20.304 |
|  |  | logarithmic function | 0.470 | 22.517 |
|  | NDVI*_GS0709_* | liner regression | 0.520 | 20.430 |
|  |  | power exponent function | 0.600 | 20.625 |
|  |  | exponential function | 0.590 | 20.090 |
|  |  | logarithmic function | 0.480 | 22.796 |

**Appendix S6** Accuracy assessments based on the remaining 25% of field-observed ANPP data of the 52 regression models developed in the Sub-regions Integrated Scheme for three sub-regions. $R^{2}$ and $RMSE$ denote the coefficient of determination and the root mean error, respectively. NDVI*_max_* represents the annual maximum NDVI. NDVI*_GS0410_*, NDVI*_GS0510_*, NDVI*_GS0610_*, NDVI*_GS0710_*, NDVI*_GS0409_*, NDVI_GS_*_0509_,* NDVI_GS_*_0609_*, NDVI_GS_*_0408_*, NDVI_GS_*_0508_*, NDVI*_GS0608_*, NDVI*_GS0708_*, and NDVI*_GS0709_* respectively denote the averaged NDVI of period from April to October, from May to October, from June to October, from July to October, from April to September, from May to September, from June to September, from April to August, from May to August, from June to August, from July to August, and from July to September.

|  | | **Forms of regression models** | **BKSSR** | | **MPSSR** | | **TPSSR** | |
| --- | --- | --- | --- | --- | --- | --- | --- | --- |
|  |  |  | 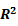 | 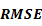 | 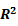 | 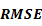 | 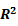 | 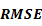 |
| **Maximum annual NDVI** | NDVI*_max_* | Liner regression | 0.540 | 11.697 | 0.511 | 18.727 | 0.597 | 22.406 |
|  |  | Power exponent function | 0.649 | 13.756 | 0.600 | 19.505 | 0.667 | 22.059 |
|  |  | Exponential function | 0.610 | 12.867 | 0.585 | 19.288 | 0.671 | 21.406 |
|  |  | Logarithmic function | 0.490 | 15.433 | 0.473 | 20.039 | 0.538 | 26.158 |
| **Averaged growing season NDVI** | NDVI*_GS0410_* | Liner regression | 0.470 | 12.926 | 0.423 | 19.232 | 0.569 | 22.062 |
|  |  | Power exponent function | 0.570 | 11.362 | 0.503 | 20.677 | 0.658 | 22.287 |
|  |  | Exponential function | 0.550 | 11.667 | 0.473 | 19.835 | 0.644 | 21.867 |
|  |  | Logarithmic function | 0.440 | 14.897 | 0.410 | 20.039 | 0.527 | 24.943 |
|  | NDVI*_GS0510_* | Liner regression | 0.470 | 13.131 | 0.464 | 18.966 | 0.569 | 22.095 |
|  |  | Power exponent function | 0.570 | 11.435 | 0.542 | 20.166 | 0.658 | 22.238 |
|  |  | Exponential function | 0.540 | 11.721 | 0.518 | 21.005 | 0.647 | 21.732 |
|  |  | Logarithmic function | 0.440 | 14.880 | 0.440 | 20.263 | 0.524 | 25.033 |
|  | NDVI*_GS0610_* | Liner regression | 0.460 | 13.474 | 0.481 | 18.775 | 0.578 | 22.377 |
|  |  | Power exponent function | 0.550 | 11.360 | 0.559 | 19.830 | 0.661 | 22.434 |
|  |  | Exponential function | 0.530 | 11.577 | 0.538 | 21.822 | 0.655 | 23.439 |
|  |  | Logarithmic function | 0.420 | 14.890 | 0.452 | 20.257 | 0.661 | 25.426 |
|  | NDVI*_GS0710_* | Liner regression | 0.422 | 13.394 | 0.481 | 18.868 | 0.564 | 22.982 |
|  |  | Power exponent function | 0.510 | 11.174 | 0.650 | 21.974 | 0.652 | 23.088 |
|  |  | Exponential function | 0.500 | 11.537 | 0.547 | 19.949 | 0.646 | 22.577 |
|  |  | Logarithmic function | 0.390 | 14.816 | 0.451 | 20.560 | 0.515 | 25.902 |
|  | NDVI*_GS0409_* | Liner regression | 0.460 | 11.611 | 0.442 | 19.052 | 0.578 | 21.882 |
|  |  | Power exponent function | 0.580 | 11.690 | 0.522 | 20.366 | 0.664 | 21.978 |
|  |  | Exponential function | 0.550 | 11.863 | 0.495 | 21.067 | 0.653 | 21.507 |
|  |  | Logarithmic function | 0.440 | 15.050 | 0.424 | 20.161 | 0.534 | 24.839 |
|  | NDVI*_GS0509_* | Liner regression | 0.460 | 13.124 | 0.481 | 18.854 | 0.572 | 21.890 |
|  |  | Power exponent function | 0.570 | 11.757 | 0.453 | 19.913 | 0.661 | 21.923 |
|  |  | Exponential function | 0.540 | 11.830 | 0.538 | 20.191 | 0.650 | 21.401 |
|  |  | Logarithmic function | 0.440 | 15.031 | 0.453 | 20.056 | 0.530 | 24.895 |
|  | NDVI*_GS0609_* | Liner regression | 0.450 | 13.425 | 0.498 | 18.717 | 0.580 | 22.157 |
|  |  | Power exponent function | 0.550 | 11.681 | 0.580 | 19.631 | 0.664 | 22.110 |
|  |  | Exponential function | 0.530 | 11.825 | 0.559 | 19.774 | 0.656 | 21.552 |
|  |  | Logarithmic function | 0.430 | 15.013 | 0.466 | 20.001 | 0.533 | 25.284 |
|  | NDVI*_GS0408_* | Liner regression | 0.480 | 13.028 | 0.410 | 19.286 | 0.589 | 21.508 |
|  |  | Power exponent function | 0.590 | 12.042 | 0.493 | 20.695 | 0.659 | 21.645 |
|  |  | Exponential function | 0.560 | 12.216 | 0.462 | 21.370 | 0.674 | 25.170 |
|  |  | Logarithmic function | 0.460 | 15.857 | 0.400 | 20.215 | 0.538 | 24.242 |
|  | NDVI*_GS0508_* | Liner regression | 0.480 | 13.078 | 0.468 | 19.014 | 0.582 | 21.514 |
|  |  | Power exponent function | 0.590 | 11.799 | 0.547 | 20.170 | 0.669 | 21.476 |
|  |  | Exponential function | 0.560 | 12.021 | 0.522 | 20.470 | 0.657 | 20.853 |
|  |  | Logarithmic function | 0.450 | 15.018 | 0.443 | 20.085 | 0.537 | 24.436 |
|  | NDVI*_GS0608_* | Liner regression | 0.480 | 13.349 | 0.487 | 18.858 | 0.595 | 21.760 |
|  |  | Power exponent function | 0.570 | 11.629 | 0.569 | 19.845 | 0.674 | 21.624 |
|  |  | Exponential function | 0.540 | 11.964 | 0.545 | 20.077 | 0.667 | 20.970 |
|  |  | Logarithmic function | 0.440 | 14.031 | 0.458 | 20.974 | 0.543 | 25.194 |
|  | NDVI*_GS0708_* | Liner regression | 0.423 | 12.999 | 0.493 | 19.069 | 0.584 | 22.442 |
|  |  | Power exponent function | 0.518 | 11.332 | 0.585 | 19.941 | 0.669 | 22.277 |
|  |  | Exponential function | 0.490 | 11.438 | 0.564 | 19.929 | 0.665 | 21.675 |
|  |  | Logarithmic function | 0.397 | 14.609 | 0.462 | 20.349 | 0.531 | 25.361 |
|  | NDVI*_GS0709_* | Liner regression | 0.410 | 13.264 | 0.506 | 18.803 | 0.567 | 22.797 |
|  |  | Power exponent function | 0.513 | 11.518 | 0.595 | 19.537 | 0.656 | 22.752 |
|  |  | Exponential function | 0.483 | 11.466 | 0.575 | 19.355 | 0.649 | 22.235 |
|  |  | Logarithmic function | 0.392 | 14.879 | 0.474 | 20.237 | 0.521 | 25.841 |

**Appendix S7** MAP, MAT and ANPP values for different grassland types in the EASR. ANPP denotes the aboveground net primary productivity per year and per square meter. MAP denotes the mean annual precipitation, and MAT denotes the mean annual temperature.

| Grassland types | MAP（mm） | MAT (℃） | ANPP（g C m^-2^ yr^-1^） |
| --- | --- | --- | --- |
| Desert steppe | 241.95 | 7.38 | 27.69 |
| Typical steppe | 319.87 | 3.53 | 47.62 |
| Meadow steppe | 455.27 | 6.20 | 56.30 |
| Meadow | 335.05 | -0.21 | 75.74 |
| Alpine meadow | 425.65 | -1.13 | 51.11 |
| Alpine steppe | 372.00 | -3.28 | 33.93 |

**Appendix S8** Categories of climate condition based on *CI_Köppen_.* (Quan *et al.*, 2013)_._ *CI_Köppen_* represents the climate index proposed by *Köppen* (1923).

|  | Hyper-arid | Arid | Semi-arid | Semi-Humid | Humid |
| --- | --- | --- | --- | --- | --- |
| *CI_köppen_* | ＜0.9 | 0.9-5.7 | 5.7-13.6 | 13.6-15.6 | ＞15.6 |

**Appendix S9** Comparison between the estimated annual ANPP values (g C m-2 yr-1) reported in this study and values reported in previous studies for different grassland types in Inner Mongolian temperate and Tibetan Plateau alpine grassland areas.

| Region | periods | Desert steppe | Typical steppe | Meadow steppe | Alpine meadow | Alpine steppe | Reference |
| --- | --- | --- | --- | --- | --- | --- | --- |
| Inner Mongolian temperate grasslands | 1998-2007 | 19.55 | 49.16 | 105.62 |  |  | Guo *et al.* (2012) |
|  |  | 27.39 | 75.21 | 129.93 |  |  | Hu *et al.* (2007) |
|  | 2001-2011 | 15.15 | 27.02 | 46.59 |  |  | Gao *et al.* (2013) |
|  | 2002-2005 | 25.47 | 60.03 | 88.52 |  |  | Ma *et al.* (2008) |
| Tibetan Plateau alpine grasslands | 2001-2004 |  |  |  | 40.86 | 22.55 | Yang *et al.* (2009) |
|  | 2011-2012 |  |  |  | 39.26 | 19.53 | Jiang *et al.* (2015) |
| Grasslands in Northern China | 2001-2005 | 27.18 | 57.42 | 87.12 | 45.18 | 18.23 | Yang *et al.* (2010) |
|  | 1982-2006 | 26.36 | 49.16 | 98.36 | 47.24 | 20.72 | Ma *et al.* (2010) |
|  | **1982-2013** | **26.72** | **50.05** | **74.81** | **55.40** | **24.66** | **This study** |

**Appendix S10** ANPP and TANPP of different grassland ecosystems around the world. ANPP denotes the aboveground net primary productivity per year and per square meter. TANPP denotes the regional total aboveground net primary productivity per year.

| Region | Area (10^7^Km^2^) | ANPP | TANPP | Reference |
| --- | --- | --- | --- | --- |
| Global | 3.23 | 143.50 | 4635.00 | Bazilevich *et al.* (1971) |
| Global | 2.40 | 127.50 | 3060.00 | Whittaker & Likens (1975) |
| Global | 2.40 | 59.38 | 1423.14 | Parton *et al.* (1993; 1995) |
| Global | 1.47 | 71.40 | 1051.71 | Xia *et al.* (2014) |
| Global |  | 72.90 |  | Yang *et al.* (2008) |
| North America | 0.37 | 109.89 | 411.00 | Lauenroth (1979) |
| North America | 0.28 | 75.10 | 207.72 | Xia *et al.* (2014) |
| South America | 0.13 | 113.40 | 141.97 | Xia *et al.* (2014) |
| Africa | 0.27 | 68.50 | 187.49 | Xia *et al.* (2014) |
| **Eurasian steppe** | **0.87** | **43.78** | **378.97** | **This study** |

# REFERENCES

Bao, G., Bao, Y.H., Qin, Z.H., Xin, X.P., Bao, Y.L., Bayarsaikan, S., Zhou, Y. & Chuntai, B. 2016. Modeling net primary productivity of terrestrial ecosystems in the semi-arid climate of the Mongolian Plateau using LSWI-based CASA ecosystem model. *International Journal of Applied Earth Observation and Geoinformation*, **46**, 84-93.

Editorial Committee of Vegetation Map of China Chinese Academy of Sciences 2007. *Vegetation map of The People’s Republic of China (1:1000 000)*. Geological Publishing House, Beijing (in Chinese).

Köppen, W. 1923. *Die Klimate der Erde*. Walter de Gruyter & Co., Berlin und Leipzig.

Olson, D.M., Dinerstein, E., Wikramanayake, E.D., Burgess, N.D., Powell, G.V.N., Underwood, E.C., D'Amico, J.A., Itoua, I., Strand, H.E., Morrison, J.C., Loucks, C.J., Allnutt, T.F., Ricketts, T.H., Kura, Y., Lamoreux, J.F., Wettengel, W.W., Hedao, P. & Kassem, K.R. 2001. Terrestrial ecoregions of the worlds: A new map of life on Earth. *Bioscience*, **51**, 933-938.

Quan, C., Han, S., Utescher, T., Zhang, C.H. & Liu, Y.S.C. 2013. Validation of temperature–precipitation based aridity index: Paleoclimatic implications. *Palaeogeography Palaeoclimatology Palaeoecology*, **386**, 86-95.

1. The map of spatial distribution of grassland types in the Eurasian steppe region was drawn according to the Vegetation map of The People’s Republic of China at 1:1,000,000 scale (Editorial Committee of Vegetation Map of China Chinese Academy of Sciences, 2007), the vegetation Map of the Mongolian Plateau reported in the study by Bao *et al.* (2016), and a Map of Terrestrial Ecoregions of the World by Olson et al. (2001) [↑](#footnote-ref-1)
