# Supplementary material for: Analysis of spatial and temporal patterns of aboveground net primary productivity in the Eurasian steppe region from 1982 to 2013
Source: Ecol Evol. 2017 Jun 6;7(14):5149–62. doi: 10.1002/ece3.3027 (PMC5528232; doi:10.1002/ece3.3027)
Supplement: Supplementary file 2 [file ECE3-7-5149-s002.docx]

# APPENDIX S11 – Data sources for field-observed data from published papers in this study

Awaya, Y., Kodani, E., Tanaka, K., Liu, J.Y., Zhuang, D.F. & Meng, Y.Q. 2004. Estimation of the global net primary productivity using NOAA images and meteorological data: changes between 1988 and 1993. *International Journal of Remote Sensing*, **25**, 1597-1613.

Bai, C.H. 2008. *Effects of land use pattern and grazing schedule on C and N reserves in 3 grassland communities in Inner Mongolian steppe*. Institute of Botany, The Chinese Academy of Sciences, Beijing (in Chinese).

Bai, Y.F. 1999. Influence of seasonal distribution of precipitation on primary productivity of Stipa krylovii community. *Acta Phytoecologica Sinica*, **23**, 155-160 (in Chinese).

Bai, Y.F., Xu, Z.X. & Li, D.X. 1994. Study on seasonal fluctuations of biomass for Leymus chinensis grassland. *Grassland of China*, **14**, 1-5 (in Chinese).

Bai, Y.F., Li, L.H., Wang, Q.B., Zhang, L.X., Zhang, Y. & Chen, Z.Z. 2000. Changes in plant species diversity and productivity along gradients of precipitation and elevation in the Xilin River basin, Inner Mongolia. *Acta Phytoecologica Sinica*, **24**, 667-673 (in Chinese).

Bai, Y.F., Wu, J.G., Xing, Q., Pan, Q.M., Huang, J.H., Yang, D.L. & Han, X.G. 2008. Primary production and rain use efficiency across a precipitation gradient on the Mongolia Plateau. *Ecology*, **89**, 2140-2153.

Bai, Y.F., Wu, J.G., Clark, C.M., Pan, Q.M., Zhang, L.X., Chen, S.P., Wang, Q.B. & Han, X.G. 2012. Grazing alters ecosystem functioning and C:N:P stoichiometry of grasslands along a regional precipitation gradient. *Journal of Applied Ecology*, **49**, 1204–1215.

Bao, L.D., Xu, W.F., Wang, Z.B., Wang, J. & Han, G.D. 2012. Carbon sequestration of Stipa breviflora desert steppe under different grazing intensities. *Journal of Inner Mongolia Agricultural University*, **33**, 94-99 (in Chinese).

Box, E.O., Holben, B.N. & Kalb, V. 1989. Accuracy of the AVHRR vegetation index as a predictor of biomass, primary productivity and net CO2 flux. *Plant Ecology*, **80**, 71-89.

Cao, G.M., Tang, Y.H., Mo, W.H., Wang, Y.S., Li, Y.N. & Zhao, X.Q. 2004. Grazing intensity alters soil respiration in an alpine meadow on the Tibetan plateau. *Soil Biology & Biochemistry*, **36**, 237-243.

Cao, G.M., Long, R.J., Zhang, F.W., Li, Y.K., Lin, L., Guo, X.W., Han, D.R. & Li, J. 2010. A method to estimate carbon storage potential in alpine Kobresia meadows on the Qinghai-Tibetan Plateau. *Acta Ecologica Sinica*, **30**, 6591-6597 (in Chinese).

Cao, J.J., Yeh, E.T., Holden, N.M., Yang, Y.Y. & Du, G.Z. 2013. The effects of enclosures and land-use contracts on rangeland degradation on the Qinghai–Tibetan plateau. *Journal of Arid Environments*, **97**, 3-8.

Chang, B.W., Jiang, Y., Hua, Y.D. & Bai, Y. 1988. Dynamics of productivity in sandy grassland community. *Grassland of China*, **8**, 33-37 (in Chinese).

Chen, F.R. 2012. *Research on the influences of disturbances types on vegetation and soil in the typical steppe of Loess region*. Institute of soil and water conservation, The Chinese Academy of Sciences, Shanxi (in Chinese).

Chen, F.R., Cheng, J.M., Liu, W., Zhu, R.B., Yang, X.M., Zhao, X.Y. & Su, J.S. 2013a. Effects of different disturbances on diversity and biomass of communities in the typical steppe of loess region. *Acta Ecologica Sinica*, **33**, 2856-2866 (in Chinese).

Chen, G.Y. 1987a. Seasonal dynamics of aboveground biomass in natural grassland. *Pratacultural Science of China*, **4**, 47-51 (in Chinese).

Chen, G.Y. 1987b. Initially search of seasonal dynamics of aboveground biomass in natural grassland. *Pratacultural Science of China*, **4**, 47-51 (in Chinese).

Chen, J., Cao, J.J., Wen, Y.L., Zhang, B.C., Zhu, B.W. & Ma, Z.T. 2014. Primary study on the allocation pattern of grassland biomass under soil water gradient of Bird Island in Qinghai Lake. *Agricultural Research in the Arid Areas*, **32**, 202-208 (in Chinese).

Chen, J.H., Zu, Y.G., Ni, H.W. & Wang, K. 1998. The growing law of aboveground biomass of main plant communities in grazing field of the Songnen grasslands. *Journal of Northeast Forestry University*, **26**, 50-53 (in Chinese).

Chen, M.D., Huang, X.D., Hou, X.M., Feng, Q.S., Yu, H., Guo, Z.G. & Liang, T.G. 2013b. Dynamic monitoring of biomass and vegetation coverage in rodent damaged grassland regions of Qinghai Province, China. *Acta Pratacurae Sinica*, **22**, 247-256 (in Chinese).

Chen, W.W., Wolf, B., Zheng, X.H., Yao, Z.S., Butterbach-Bahl, K., Brüggemann, N., Liu, C.Y., Han, S.H. & Han, X.G. 2011. Annual methane uptake by temperate semiarid steppes as regulated by stocking rates, aboveground plant biomass and topsoil air permeability. *Global Change Biology*, **17**, 2803–2816.

Chen, Y.P., Li, Y.Q., Zhao, X.Y., Awada, T., Shang, W. & Han, J.J. 2012. Effects of grazing exclusion on soil properties and on ecosystem carbon and nitrogen storage in a sandy rangeland of Inner Mongolia, northern China. *Environmental Management*, **50**, 622-32.

Chen, Y.X., Lee, G., Lee, P. & Oikawa, T. 2007. Model analysis of grazing effect on above-ground biomass and above-ground net primary production of a Mongolian grassland ecosystem. *Journal of Hydrology*, **333**, 155-164.

Chen, Z.Z., Huang, D.H. & Li, J.S. 1988. A preliminary study on the biomass dynamics of the stipa breviflora steppe in Wulanchabu League of Inner Mongolia. *Journal of Arid Land Resources and Environment*, **2**, 63-70 (in Chinese).

Cheng, J., Wu, G.L., Zhao, L.P., Li, Y., Li, W. & Cheng, J.M. 2011a. Cumulative effects of 20-year exclusion of livestock grazing on above-and belowground biomass of typical steppe communities in arid areas of the Loess Plateau, China. *Plant Soil and Environment*, **57**, 40-44.

Cheng, J.M. 1986. Biomass of grassland at Yunwu Mountain, Guyuan County. *Grassland and Forage in China*, **3**, 21-25 (in Chinese).

Cheng, J.M., Cheng, J. & Yang, X.M. 2011b. Grassland vegetation and soil carbon sequestration in the Loess Plateau. *Journal of Natural Resources*, **26**, 401-411 (in Chinese).

Cheng, J.M., Zhou, H.Y., Peng, X.L. & Jia, H.Y. 1993. Dynamics of community structure and biomass of Stipa bungeana grassland under closed conditions. *Pratacultural Science of China*, **10**, 48-52 (in Chinese).

Cheng, J.M., Jing, Z.B., Jin, J.W. & Gao, Y. 2014. Restoration and utilization mechanism of degraded grassland in the semi-arid region of Loess Plateau. *Scientia SinicaVitae.*, **44**, 267-279 (in Chinese).

Daojier, L.B.S., Sun, C.Z., Chen, Z.Z. & Huang, D.H. 1990. The dynamic of biomass and relationship between biomass and precipitation of desert steppe in Inner Mongolia. *Arid Land Geography*, **13**, 10-17 (in Chinese).

Deng, L., Zhang, Z.N. & Shangguan, Z.P. 2014a. Long-term fencing effects on plant diversity and soil properties in China. *Soil & Tillage Research*, **137**, 7-15.

Deng, L., Sweeney, S. & Shangguan, Z.P. 2014b. Grassland responses to grazing disturbance: plant diversity changes with grazing intensity in a desert steppe. *Grass and Forage Science*, **69**, 524–533.

Deng, L., Wang, K.B., Li, J.P., Shangguan, Z.P. & Sweeney, S. 2014c. Carbon Storage Dynamics in Alfalfa Medicago sativa . Fields in the Hilly-Gully Region of the Loess Plateau, China. *CLEAN - Soil Air Water*, **42**, 1253–1262.

Ding, X.H., Luo, S.Z., Liu, J.W., Li, K. & Liu, G.H. 2012a. Longitude gradient changes on plant community and soil stoichiometry characteristics of grassland in Hulunbeir. *Acta Ecologica Sinica*, **32**, 3467-3476 (in Chinese).

Ding, X.H., Gong, L., Wang, D.B., Wu, X. & Liu, G.H. 2012b. Grazing effects on eco-stoichiometry of plant and soil in Hulunbeir, Inner Mogolia. *Acta Ecologica Sinica*, **32**, 4722-4730 (in Chinese).

Dong, K.H., Jin, Z.L., Wang, Y.K. & Zhang, J.Q. 1995. A study on dynamics of herbage yield in Bothriochloa Ischaemum shrub grassland in Shanxi. *Grassland of China*, 13-16, 21 (in Chinese).

Dong, Q.M., Zhao, X.Q., Ma, Y.S., Shi, J.J., Wang, Y.L., Li, S.X., Yang, S.H., Wang, L.Y. & Sheng, L. 2012. Influence of grazing on biomass, growth ratio and compensatory effect of different plant groups in Kobresia parva meadow. *Acta Ecologica Sinica*, **32**, 2640-2650 (in Chinese).

Dong, X.Y., Fu, H., Li, X.D., Niu, D.C., Guo, D. & Li, X.D. 2010. Effects on plant biomass and CNP contents of plants in grazed and fenced steppe grassland of the Loess Plateau. *Acta Prataculturae Sinica*, **19**, 175-182 (in Chinese).

Du, F., Liang, Z.S., Xu, X.X., Shan, L. & Zhang, X.C. 2007. The community biomass of abandoned farmland and its effects on soil nutrition in the Loess Hilly Region of Northern Shaanxi,China. *Acta Ecologica Sinica*, **27**, 1673-1683 (in Chinese).

Fan, Y.G., Hu, Y.K., Li, K.H., Yu, J.M. & Wang, X. 2008. Effect of different disturbances on the diversity and biomass of the Phytobiocoenoses in alpine steppes. *Arid Zone Research*, **25**, 531-536 (in Chinese).

Fan, Y.J., Hou, X.Y., Shi, H.X. & Shi, S.L. 2013. Effects of grazing and fencing on carbon and nitrogen reserves in plants and soils of alpine meadow in the three headwater resource regions. *Russian Journal of Ecology*, **44**, 80-88.

Fan, Y.M., Wu, H.Q., Jin, G.L., Liu, W. & A de, L.T. 2012. Change of plant community character and soil nutrients under different utilization models of spring-autumn steppe. *Xinjiang Agricultural Sciences*, **49**, 1503-1508 (in Chinese).

Fang, K., Song, N.P., Wei, L. & An, H. 2012. The effect of different grazing systems on aboveground biomass and interspecific relationships in desert steppe. *Acta Prataculturae Sinica*, **21**, 12-22 (in Chinese).

Ganju, R., Guo, Y.Q., Gao, Q.Z., Duan, M.J., Wan, Y.F., Li, Y.E. & DanJiu, B.L. 2013. A study on optimal grazing rates in Stipapurpurea alpine grassland in Northern Tibet. *Acta Prataculturae Sinica*, **22**, 130-137 (in Chinese).

Gao, N.N., Chen, J., Zhang, P.L., Liu, S.J., Xu, Y.F. & Hu, T.M. 2014. Effects of grazing intensity on the spatial distribution of aboveground biomass of alpine Kobresia meadow in Tibetan. *Acta Agrestia Sinica*, **22**, 255-260 (in Chinese).

Gao, Y.H., Luo, P., Wu, N., Chen, H. & Wang, G.X. 2007. Grazing Intensity Impacts on Carbon Sequestration in an Alpine Meadow on the Eastern Tibetan Plateau. *Research Journal of Agriculture and Biological Sciences*, **3**, 642-647.

Gao, Y.Z., Giese, M., Lin, S., Sattelmacher, B., Zhao, Y. & Brueck, H. 2008. Belowground net primary productivity and biomass allocation of a grassland in Inner Mongolia is affected by grazing intensity. *Plant and Soil*, **307**, 41-50.

Geng, Y., Wang, Y.H., Yang, K., Wang, S.P., Zeng, H., Baumann, F., Kuehn, P., Scholten, T. & He, J.S. 2012. Soil respiration in Tibetan alpine grasslands: belowground biomass and soil moisture, but not soil temperature, best explain the large-scale patterns. *Plos One*, **7**, : e34968.

Gong, X., Brueck, H., Giese, K.M., Zhang, L., Sattelmacher, B. & Lin, S. 2008. Slope aspect has effects on productivity and species composition of hilly grassland in the Xilin River Basin, Inner Mongolia, China. *Journal of Arid Environments*, **72**, 483-493.

Gu, W.R., Zhang, X.H., Zhu, J.Z., Sun, Z.J., Mu, X.Y. & Wang, X.J. 2013. Impact of seasonal rest grazing on plant community quantity characteristics under different grazing intensities. *Xinjiang Agricultural Sciences*, 1145-1149 (in Chinese).

Guo, Y.J., Han, L., Li, G.D., Han, J.G., Wang, G.L., Li, Z.Y. & Wilson, B. 2012. The effects of defoliation on plant community, root biomass and nutrient allocation and soil chemical properties on semi-arid steppes in northern China. *Journal of Arid Environments*, **78**, 128-134.

Ha, Q. 2012. *The carbon storage of different grassland patterns in Saihanwula national nature reserve*. Inner Mongolia Agricultural University, Inner Mongolia (in Chinese).

Han, B., Fan, J.W. & Zhong, H.P. 2006. Grassland biomass of communities along gradients of the Inner Mongolia grassland transect. *Journal of Plant Ecology*, **30**, 553-562 (in Chinese).

Han, D.R. 2012. *The carbon storage of alpine grassland in Qinghai-Tibetan plateau and its relationship to the climatic factors*. Northwest institute of plateau biology, The Chinese Academy of Sciences, Qinghai (in Chinese).

Han, G.D., Hao, X.Y., Zhao, M.L., Wang, M.J., Ellert, B.H., Willms, W. & Wang, M. 2008. Effect of grazing intensity on carbon and nitrogen in soil and vegetation in a meadow steppe in Inner Mongolia. *Agriculture Ecosystems & Environment*, **125**, 21-32.

Han, L., Guo, Y.J., Han, J.G., Guo, Y.J. & Tang, H. 2010. A study on the diversity and aboveground biomass in a Leymus chinensis meadow steppe community under different cutting intensities. *Acta Prataculture Sinica*, **19**, 70-75 (in Chinese).

Han, W.J., Hou, X.Y., Olokhnuud, C.L. & Schellenberg, M.P. 2014. The Characteristics of Plant Communities Along East Eurasian Steppe Transect. *Journal of Integrative Agriculture*, **13**, 1157-1164.

Han, X.W., Tsunekawa, A., Tsubo, M. & Li, S.Q. 2011. Aboveground biomass response to increasing nitrogen deposition on grassland on the northern Loess Plateau of China. *Acta Agriculturae Scandinavica, Section B - Soil and Plant Science*, **61**, 112-121.

Hayashi, I., Kawada, K., Akimova, A. & Nakamura, T. 2006. Floristic composition and plant biomass of the grasslands in the vicinity of Pavlodar, Kazakhstan. *Grassland Science*, **52**, 141-146.

Hayashi, I., Kawada, K., Kurosu, M., Batjargal, A., Tsundeekhuu, T. & Nakamura, T. 2008. Grazing Effects on Floristic Composition and Above Ground Plant Biomass of the Grasslands in the Northeastern Mongolian Steppes. *Journal of Ecology and Environment*, **31**, 115-123.

He, H.Y., Su, J.Q., Huang, L., He, R.L. & Li, X.R. 2011. Effects of fire on the structure of herbage synusia vegetation in desertified steppe, North China. *Acta Ecologica Sinica*, **11**, 364-370 (in Chinese).

He, N.P., Yu, Q., Wu, L., Wang, Y.S. & Han, X.G. 2009. Carbon and nitrogen store and storage potential as affected by land-use in a Leymus chinensis grassland of northern China. *Soil Biology & Biochemistry*, **40**, 2952-2959.

Hu, E.C. 2009. *Study on the dynamics and relationship between the standing live aboveground biomass and stocking rate in different scales in the steppe grassland*. Inner Mongolia University, Inner Mongolia (in Chinese).

Hu, Z.Z., Sun, J.X., Zhang, Y.S., Xu, C.L. & Zhang, Z.H. 1988a. Primary production in Tianzhu alpine Polygonum viviparum meadow: biomass dynamics and conversion efficiency for solar radiation. *Acta Phytoecologica et Geobotanica Sinica*, **12**, 123-133 (in Chinese).

Hu, Z.Z., Sun, J.X., Zhang, Y.S., Xu, C.L. & Zhang, Z.H. 1988b. Studies on matter production and efficiency on energy in Tianzhu alpine Kobresia capillifolia grassland: 1. Structure characteristics of community and dynamics of phytomass. *Pratacultural Science of China*, **5**, 7-13 (in Chinese).

Hu, Z.Z., Sun, J.X., Li, Y., Long, R.J. & Yang, F.L. 1994. The characteristics of biomass and conversion efficiency of solar radiation for principal types of alpine grasslands in Tianzhu, Gansu Province. *Acta Phytoecologica Sinica*, **18**, 121-131 (in Chinese).

Huang, D.H., Chen, Z.Z. & Zhang, H.F. 1986. A comparative study on biomass of root systems of different grassland in middle reaches of Xilin River, Inner Mongolia. *Journal of Sichuan Grassland*, 55-78 (in Chinese).

Huang, D.Q., Yu, L., Zhang, Y.S. & Zhao, X.Q. 2011. Belowground biomass and its relationship to environmental factors of natural grassland on the northern slopes of the Qilian Mountains. *Acta Prataculturae Sinica*, **20**, 1-10 (in Chinese).

Iijima, Y., Kawaragi, T., Ito, T., Akshalov, K., Tsunekawa, A. & Shinoda, M. 2008. Response of plant growth to surface water balance during a summer dry period in the Kazakhstan steppe. *Hydrological Processes*, **22**, 2974-2981.

Ji, S.J., Yan, G., Li, D.F. & Wang, G.H. 2009. Plant coverage is more important than species richness in enhancing aboveground biomass in a premature grassland, northern China. *Agriculture Ecosystems & Environment*, **129**, 491-496.

Jiang, J.S., Hu, Z.Z. & Zhu, X.Y. 1995. Studies on primary production and the Relationship between primary production and the major ecological factors of Puccinellia Grassland. *Grassland of China*, 16-22 (in Chinese).

Jin, Y.X., Xu, B., Yang, X.C., Li, J.Y., Ma, H.L., Gao, T. & Yu, H.D. 2013. Below-ground biomass and features of environmental factors in the degree of grassland desertification. *Acta Prataculture Sinica*, **22**, 44-51 (in Chinese).

Jing, Z.B., Cheng, J.M., Su, J.S., Bai, Y. & Jin, J.W. 2014. Changes in plant community composition and soil properties under 3-decade grazing exclusion in semiarid grassland. *Ecological Engineering*, **64**, 171-178.

Kinugasa, T., Tsunekawa, A. & Shinoda, M. 2012. Increasing nitrogen deposition enhances post-drought recovery of grassland productivity in the Mongolian steppe. *Oecologia*, **170**, 857-65.

Li, D.M., Jiao, F., Lei, B. & Zhang, Z. 2014a. Aboveground biomass production and soil moisture characteristics of different herb communities in the Loess Hilly-gully region. *Science of Soil and Water Conservation*, **12**, 33-37 (in Chinese).

Li, F., Yu, P.J., Shen, X.J., Song, Y.T., Li, Q., Zhang, H.Y. & Zhou, D.W. 2014b. Community productivity and soil carbon sequestration after Melilotus of ficinalis and Medicago falcata reseeding on degraded grassland. *Pratacultural Science of China*, **31**, 361-366 (in Chinese).

Li, G., Li, Y.G., Liu, M.Z. & Jiang, G.M. 2011a. Biomass carbon storage and net primary production in different habitats of Hunshandake Sandland, China. *Science & Technology Review*, **29**, 30-37 (in Chinese).

Li, G.D. 1990. The study of underground Phytomass and Its Seasonal Changes in Alpine Grass-Kobresia meadow of Tianzhu Area, Gansu. *Acta Prataculturae Sinica*, **1**, 42-46 (in Chinese).

Li, G.T., Zhang, M.Z., Zhang, B.Z. & Zhou, S.Y. 1995. A study on biomass of cakcayr forest. *Journal of Inner Mongolia Forestry College*, **17**, 35-43 (in Chinese).

Li, K.H., Hu, Y.K., Wang, X., Fang, Y.G. & Wumaier, W.S. 2007a. Relationship between aboveground biomass and environmental factors along an altitude gradient of alpine grassland. *Chinese Journal of Applied Ecology*, **18**, 2019-2024 (in Chinese).

Li, N., Wang, G.X., Yang, Y., Gao, Y.H., Liu, L.A. & Liu, G.S. 2011b. Short-term effects of temperature enhancement on community structure and biomass of alpine meadow in the Qinghai-Tibet Plateau. *Acta Ecologica Sinica*, **31**, 895-905 (in Chinese).

Li, Q., Zhou, D.W., Jin, Y.H., Wang, M.L., Song, Y.T. & Li, G.D. 2014c. Effects of fencing on vegetation and soil restoration in a degraded alkaline grassland in northeast China. *Journal of Arid Land*, **6**, 478-487.

Li, Q., Yang, J., Song, B.Y., Ma, W.H., Zhao, L.Q., Zhang, L.X. & Hou, H. 2014d. The impacts of different enclosure durations on degraded Stipa grandis grassland productivity and soil carbon and nitrogen storage. *Chinese Journal of Ecology*, **33**, 896-901 (in Chinese).

Li, T. 2013a. *Research on soil and vegetation carbon storage of grassland under different land use and degree in Aba Pastoral Areas*. Sichuan Agricultural University, Sichuan (in Chinese).

Li, W., Huang, H.Z., Zhang, Z.N. & Wu, G.L. 2011c. Effects of grazing on the soil properties and C and N storage in relation to biomass allocation in an alpine meadow. *Journal of Soil Science and Plant Nutrition*, **11**, 27-39.

Li, W.J., Wang, Z., Han, Q.F., Ren, Z.H., Yan, M.K., Zhang, P., Jia, Z.K. & Yang, B.P. 2013a. Evaluation on carbon sequestration effects of artificial alfalfa pastures in the Loess Plateau area. *Acta Ecologica Sinica*, **33**, 7467-7477 (in Chinese).

Li, X.L., Huang, B.N., Qiao, Y.M., Sun, H.S., Li, F.J. & Sun, B.C. 1996. Biomass allocation and seeding development of Kobresia on Tibetan Plateau. *Acta Prataculturae Sinica*, **5**, 48-54 (in Chinese).

Li, Y. & Qi, Z. 1993. Primary production of alpine shrub-moss grassland in Qilian Mountains and response of soil ecosystems to disturbance. *Acta Prataculturae Sinica*, **2**, 66-74 (in Chinese).

Li, Y.H. 2005. *Grassland ecosystem production and leaf area index in the Gonghe Basin, Qinghai province*. Institute of Geographic Sciences and Natural Resources Research, The Chinese Academy of Sciences, Beijing (in Chinese).

Li, Y.H., Luo, T.X. & Lu, Q.i. 2008. Plant height as a simple predictor of the root to shoot ratio: Evidence from alpine grasslands on the Tibetan Plateau. *Journal of Vegetation Science*, **19**, 245-252.

Li, Y.J. 2013b. *Response of alpine grassland to land use pattern changes on Qinghai-Tibet Plateau*. Gansu Agricultural University, Gansu (in Chinese).

Li, Y.J. 2013c. *Effects of rest grazing on plant diversity and organic carbon storage on Stipa Baicalensis steppe in Inner Mongolia*. Shenyang Agricultural University, Shenyang (in Chinese).

Li, Y.J., Zhu, Y., Zhao, J.N., Li, G., Wang, H., Lai, X. & Yang, D.L. 2014e. Effects of Rest Grazing on Organic Carbon Storage in Stipa grandis Steppe in Inner Mongolia, China. *Journal of Integrative Agriculture*, **13**, 624-634.

Li, Y.Q., Zhou, X.H., Brandle, J.R., Zhang, T.H., Chen, Y.P. & Han, J.J. 2012. Temporal progress in improving carbon and nitrogen storage by grazing exclosure practice in a degraded land area of China's Horqin Sandy Grassland. *Agriculture Ecosystems & Environment*, **159**, 55-61.

Li, Y.Y., Shao, M.A., Zheng, J.Y. & Li, Q.F. 2007b. Impact of grassland recovery and reconstruction on soil organic carbon in the northern Loess Plateau. *Acta Ecologica Sinica*, **27**, 2279-2287 (in Chinese).

Li, Y.Y., Dong, S.K., Wen, L.u., Wang, X.X. & Wu, Y. 2013b. The effects of fencing on carbon stocks in the degraded alpine grasslands of the Qinghai-Tibetan Plateau. *Journal of Environmental Management*, **128**, 393-399.

Li, Y.Y., Dong, S.K., Wen, L., Wang, X.X. & Wu, Y. 2014f. Soil carbon and nitrogen pools and their relationship to plant and soil dynamics of degraded and artificially restored grasslands of the Qinghai-Tibetan Plateau. *Geoderma*, **213**, 178-184.

Li, Y.Y., Dong, S.K., Zhu, L., Wen, L., Li, X.Y. & Wang, X.X. 2013c. Adaptation strategies of reproduction of plant community in response to grassland degradation and artificial restoration. *Acta Ecologica Sinica*, **33**, 4683-4691 (in Chinese).

Li, Y.Z. & Li, B. 1991. Study on the biomass of Leymus chinense grassland in Xilin River Basin of Inner Mongolia. *Grassland of China*, 5-8 (in Chinese).

Li, Y.Z., Fan, J.W., Zhang, L.X., Zhai, J., Liu, G.F. & Li, J. 2013d. The impact of different land use and management on community composition, species diversity and productivity in typical temperate grassland. *Acta Prataculturae Sinica*, **22**, 1-9 (in Chinese).

Lin, Y., Hong, M., Han, G.D., Zhao, M.L., Bai, Y.F. & Chang, S.X. 2010. Grazing intensity affected spatial patterns of vegetation and soil fertility in a desert steppe. *Agriculture Ecosystems & Environment*, **138**, 282-292.

Liu, H.L., Wang, J.W., Lv, J.Y. & Wang, K. 2010. Response of grasslands conversion to croplands on soil organic carbon in Bashang area of Northern China. *African Journal of Biotechnology*, **9**, 1783-1788.

Liu, J.D. 2010. *Model and remote sensing herbage yield model-To take Evenk Autonomous banner Inner Mongolia as example*. Inner Mongolia Agricultural University, Inner Mongolia (in Chinese).

Liu, J.X. 1996. Population distribution and dynamic of primary productivity in Artemisia ordosica community. *Acta Prataculturae Sinica*, **5**, 23-29 (in Chinese).

Liu, L.L. 2006. *A study on plant diversity and biomass of managed meadows in the Tibetan region, NW Yunnan, China*. Xishuangbanna Tropical Botanical Garden, The Chinese Academy of Sciences, Yunnan (in Chinese).

Liu, M., Liu, G.H., Wu, X., Wang, H. & Chen, L. 2014a. Vegetation traits and soil properties in response to utilization patterns of grassland in Hulun Buir City, Inner Mongolia, China. *Chinese Geographical Science*, **24**, 471-478.

Liu, Y., Ma, Y.S., Li, S.X., Zheng, W. & Yang, S.H. 2014b. Species diversity and biomass characteristics of different grain-for-green grassland in the Northern region of Qinghai lake. *Acta Agriculturae Boreali-occidentalis Sinica*, **23**, 48-52 (in Chinese).

Liu, Y.Z., Xing, Q. & Jiang, Y. 1996. Dynamics of aboveground biomass in Horqin grassland. *Journal of Inner Mongolia Prataculture*, **Z2**, 36-45 (in Chinese).

Liu, Z.L. & Li, Z.H. 1987. Primary productivity of Leymus chinense and Stipa grandis steppe in Inner Mongolia. *Journal of Arid Land Resources and Environments*, **1**, 13-33 (in Chinese).

Lu, J.F., Dong, Z.B., Li, W.J. & Hu, G.Y. 2014a. The effect of desertification on carbon and nitrogen status in the northeastern margin of the Qinghai-Tibetan Plateau. *Environmental Earth Sciences*, **71**, 807-815.

Lu, X.Y., Yan, Y., Fan, J.H., Cao, Y.Z. & Wang, X.D. 2011. Dynamics of above- and below-ground biomass and C, N, P accumulation in the alpine steppe of Northern Tibet. *Journal of Mountain Science*, **8**, 838-844.

Lu, Y.H., Ma, Z.M., Zhao, Z.J., Sun, F.X. & Fu, B.J. 2014b. Effects of Land Use Change on Soil Carbon Storage and Water Consumption in an Oasis-Desert Ecotone. *Environmental Management*, **53**, 1066-1076.

Lv, X.L. 1994. Dynamics of primary productivity of meadow steppe in Hulun Buir Region. *Grassland of China*, 9-11, 8 (in Chinese).

Ma, H.B., Shen, Q.J., Xie, Y.Z. & Shen, Y. 2013. Effects of the enclosing on the underground carbon storage of typical steppe in Ningxia. *Journal of Agricultural Sciences*, **34**, 1-4 (in Chinese).

Ma, K.P., Zhou, R.C. & Zhang, R. 1993. Studies on the structure and seasonal dynamics of aboveground biomass of Calamagrostis angustifolia grassland in Sanjiang Plain. *Grassland of China*, 27-31 (in Chinese).

Ma, S.Q. 1987. Above-ground stand crop biomass of Stipa grandis and S. krylovii grasslands in Inner Mongolia. *Journal of Arid Land Resources and Environment*, **1**, 95-106 (in Chinese).

Ma, T., Wu, G.L., He, Y.L., Wen, S.J., He, J.L., Liu, J.X. & Du, G.Z. 2007. The effect of simulated mowing of the fertilizing level on community production and compensatory responses on the Qinghai-Tibetan. *Acta Ecologica Sinica*, **27**, 2288-2293 (in Chinese).

Matsushita, B. & Tamura, M. 2002. Integrating remotely sensed data with an ecosystem model to estimate net primary productivity in East Asia. *Remote Sensing of Environment*, **81**, 58-66.

Ni, J., Wang, G.H., Bai, Y.F. & Li, X.Z. 2007. Scale-dependent relationships between plant diversity and above-ground biomass in temperate grasslands, south-eastern Mongolia. *Journal of Arid Environments*, **68**, 132-142.

Niu, D., Hall, S.J., Fu, H., Kang, J., Qin, Y. & Elser, J.J. 2011. Grazing exclusion alters ecosystem carbon pools in Alxa desert steppe. *New Zealand Journal of Agricultural Research*, **54**, 127-142.

Pavlova, V.N., Varcheva, S.E., Bokusheva, R. & Calanca, P. 2014. Modelling the effects of climate variability on spring wheat productivity in the steppe zone of Russia and Kazakhstan. *Ecological Modelling*, **277**, 57-67.

Peng, H.Y., Li, X.Y. & Tong, S.Y. 2013. Effects of shrub encroachment on biomass and biodiversity in the typical steppe of Inner Mongolia. *Acta Ecologica Sinica*, **33**, 7221-7229 (in Chinese).

Peng, Y.M. 1997. Aboveground biomass and nutrient dynamics of Leymus chinense and Stipa baicalensis grasslands. *Grassland of China*, 26-29 (in Chinese).

Qi, Y., Huang, Y.M., Wang, Y., Zhao, J. & Zhang, J.H. 2011. Biomass and its allocation of four grassland species under different nitrogen levels. *Acta Ecologica Sinica*, **31**, 5121-5129 (in Chinese).

Qi, Y.C., Dong, Y.S., Jin, Z., Peng, Q., Xiao, S.S. & He, Y.T. 2010. Spatial Heterogeneity of Soil Nutrients and Respiration in the Desertified Grasslands of Inner Mongolia, China. *Pedosphere*, **20**, 655-665.

Qiu, L.P., Wei, X.R., Zhang, X.C. & Cheng, J.M. 2013. Ecosystem Carbon and Nitrogen Accumulation after Grazing Exclusion in Semiarid Grassland. *Plos One*, **8**, 268-277.

Qu, H., Zhao, X.Y., Wang, S.K., Huang, W.D. & Mao, W. 2014. Effects of different vegetation communities on soil carbon and nitrogen contents in Urad desert steppe. *Pratacultural Science*, **31**, 355-360 (in Chinese).

Ren, A.C. 2008. *Grassland biomass on North-western Plateau of Sichuan and vegetation indexes relation using landsat TM image*. Sichuan Agricultural University, Sichuan (in Chinese).

Ren, H.R., Zhou, G.S. & Zhang, X.S. 2011. Estimation of green aboveground biomass of desert steppe in Inner Mongolia based on red-edge reflectance curve area method. *Biosystems Engineering*, **109**, 385-395.

Rong, Y.P., Yuan, F. & Ma, L. 2014. Effectiveness of exclosures for restoring soils and vegetation degraded by overgrazing in the Junggar Basin, China. *Grassland Science*, **60**, 118-124.

S, R.M. 1998. Developing simple, operational, consistent NDVI-vegetation models by applying environmental and climatic information: Part I. Assessment of net primary production. *International Journal of Remote Sensing*, **19**, 97-117.

Sa, W.J., An, L.Z. & Sa, W. 2012. Changes in plant community diversity and aboveground biomass along with altitude within an alpine meadow on the Three-River source region. *Chinese Science Bulletin*, **57**, 3573-3577.

Seyin, B.Y. & Bao, M.R. 1992. Dynamics of primary productivity in plain lowland meadow grassland. *Prataculture of Inner Mongolia*, 50-56 (in Chinese).

Shen, M.G., Tang, Y.H., Klein, J., Zhang, P.C., Gu, S., Shimono, A. & Chen, J. 2008. Estimation of aboveground biomass using in situ hyperspectral measurements in five major grassland ecosystems on the Tibetan Plateau. *Journal of Plant Ecology*, **1**, 247-257.

Shen, Y.Y., Yan, S.G., Zhu, X.Y. & Zhao, Y. 1995. Primary productivity of saline pasture in Hexi Corridor. *Acta Prataculturae Sinica*, **4**, 44-50 (in Chinese).

Shi, F.S., Wu, N. & Luo, P. 2008. Effect of temperature enhancement on community structure and biomass of subalpine meadow in Northwestern Sichuan. *Acta Ecologica Sinica*, **28**, 5286-5293 (in Chinese).

Shi, F.S., Chen, H., Wu, Y. & Wu, N. 2010. Effects of livestock exclusion on vegetation and soil properties under two topographic habitats in an alpine meadow on the eastern qinghai-tibetan plateau. *Polish Journal of Ecology*, **58**, 125-133.

Song, L.L., Fan, J.W., Zhong, H.P. & Wang, N. 2010. Changes of biomass and species richness of grassland community along an altitude gradient in Hongchiba, Chongqing. *Acta Agrestia Sinica*, **18**, 160-166 (in Chinese).

Sun, D.S., Wesche, K., Chen, D.D., Zhang, S.H., Wu, G.L., Du, G.Z. & Comerford, N.B. 2011. Grazing depresses soil carbon storage through changing plant biomass and composition in a Tibetan alpine meadow. *Plant Soil and Environment*, **57**, 271-278.

Sun, S.X., Cui, Z.M., Chen, L.B., Jia, L.J. & Wei, Z.J. 2014. Effects of seasonal regulation of grazing intensity on N and C of the main plant species and soil in desert grassland. *Chinese Journal of Grassland*, **36**, 49-54 (in Chinese).

Tang, L., Dang, X.H., Liu, G.B., Shao, C.K. & Xue, S. 2014. Response of Artificial Grassland Carbon Stock to Management in Mountain Region of Southern Ningxia, China. *Chinese Geographical Science*, **24**, 436-443.

Tucker, C.J., Vanpraet, C.L., Sharman, M.J. & Ittersum, G.V. 1985. Satellite remote sensing of total herbaceous biomass production in the senegalese sahel: 1980–1984. *Remote Sensing of Environment*, **17**, 233-249.

Urano, T., Hirota, M., Ishgaldan, B., Li, S.G., Asanuma, J., Mariko, S., Sugita, M. & Oikawa, T. 2010. Effects of exclosure on aboveground biomass, vegetation constitution, and midday gross primary productivity in semi-arid Mongolian steppe. *Journal of Agricultural Meteorology*, **66**, 227-236.

Wang, C.T. 2006. *Relationship between productivity and species diversity of plant in alpine meadow*. Northwest Plateau Institute of Biology, The Chines Academy of Science, Qinghai (in Chinese).

Wang, C.T., Long, R.J., Wang, Q.L., Jing, Z.C. & Shi, J.J. 2009. Changes in plant diversity, biomass and soil C, in alpine meadows at different degradation stages in the headwater region of three rivers, China. *Land Degradation & Development*, **20**, 187-198.

Wang, C.T., Cao, G.M., Wang, Q.L., Jing, Z.C., Ding, L.M. & Long, R.J. 2008. Changes in plant biomass and species composition of alpine Kobresia meadows along altitudinal gradient on the Qinghai-Tibetan Plateau. *Science in China Series C-Life Sciences*, **51**, 86-94.

Wang, C.T., Long, R.J., Wang, G.X., Liu, W., Wang, Q.L., Zhang, L. & Wu, P.F. 2010. Relationship between plant communities, characters, soil physical and chemical properties, and soil microbiology in alpine meadows. *Acta Prataculturae Sinica*, **19**, 25-34 (in Chinese).

Wang, G.J., Wang, S.P., Hao, Y.B. & Cai, X.C. 2005. Effect of grazing on the plant functional group diversity and community biomass and their relationship along a precipitation gradient in Inner Mongolia Steppe. *Acta Ecologica Sinica*, **25**, 1649-1656 (in Chinese).

Wang, G.M., Yu, G.Y. & Su, H. 1987. The Research of biomass on different type nature grasslands. *Grassland of China*, **7**, 18-21 (in Chinese).

Wang, J. & Baoyin, T.G.T. 2014. Species composition and the aboveground biomass of Stipa glareosa community in desert steppe. *Chinese Journal of Grassland*, **36**, 108-111 (in Chinese).

Wang, J.L. 2010. *Preliminary research of plant and soil carbon sequestration potential in alpine meadow of Qinghai province*. Northwest Institute of Plateau Biology, The Chinese Academy of Science, Qinghai (in Chinese).

Wang, K.B., Li, J.P. & Shangguan, Z.P. 2012. Biomass Components and Environmental Controls in Ningxia Grasslands. *Journal of Integrative Agriculture*, **11**, 2079-2087.

Wang, Q.J. & Wang, S.M. 1993. Biomass and grazing of sheeps in sand grassland, Aohan pasture. *Prataculture of Inner Mongolia*, 1-5 (in Chinese).

Wang, Q.S. & Li, B. 1994. Preliminary study on biomass of Artemisia ordosica community in Ordos Plateau sand land of China. *Acta Phytoecologica Sinica*, **18**, 347-353 (in Chinese).

Wang, Q.Y., Wang, F.C. & Wang, Y.M. 1996. Dynamics of aboveground biomass in a dry steppe in Kailu Plain. *Prataculture of Inner Mongolia*, 46-48 (in Chinese).

Wang, X.X., Dong, S.K., Yang, B., Li, Y.Y. & Su, X.K. 2014a. The effects of grassland degradation on plant diversity, primary productivity, and soil fertility in the alpine region of Asia's headwaters. *Environmental Monitoring and Assessment*, **186**, 6903-17.

Wang, Y.F. & Jiang, S. 1982. The effect of arid climate on the community structure and aerial biomass of Stipa grandis steppe. *Acta Phytoecologica et Geobotanica Sinica*, **6**, 333-338 (in Chinese).

Wang, Z., Luo, T.X., Li, R.C., Tang, Y.H. & Du, M.Y. 2013. Causes for the unimodal pattern of biomass and productivity in alpine grasslands along a large altitudinal gradient in semi-arid regions. *Journal of Vegetation Science*, **24**, 189–201.

Wang, Z., Yun, X.J., Wei, Z.J., Schellenberg, M.P., Wang, Y.F., Yang, X. & Hou, X.Y. 2014b. Responses of Plant Community and Soil Properties to Inter-Annual Precipitation Variability and Grazing Durations in a Desert Steppe in Inner Mongolia. *Journal of Integrative Agriculture*, **13**, 1171-1182.

Wei, S.C., Li, Q.R., Zhang, F.T., Sun, S.Y. & Jiang, Y. 1986. Dynamics of production and nutrition of Stipa grandis-Cleistogenes squarrosapasture. *Grassland of China*, **6**, 10-15 (in Chinese).

Wen, L., Dong, S.K., Li, Y.Y., Wang, X.X., Li, X.Y., Shi, J.J. & Dong, Q.M. 2013. The impact of land degradation on the C pools in alpine grasslands of the Qinghai-Tibet Plateau. *Plant and Soil*, **368**, 329-340.

Wu, G.L., Ren, G.H., Dong, Q.M., Shi, J.J. & Wang, Y.L. 2014a. Above- and belowground Response along Degradation Gradient in an Alpine Grassland of the Qinghai-Tibetan Plateau. *CLEAN – Soil Air Water*, **42**, 319–323.

Wu, J.S., Zhang, X.Z., Shen, Z.X., Shi, P.L., Yu, C.Q. & Chen, B.X. 2014b. Effects of livestock exclusion and climate change on aboveground biomass accumulation in alpine pastures across the Northern Tibetan Plateau. *Chinese Science Bulletin*, **59**, 4332-4340.

Wu, L. & Zhang, X.S. 2006. Characters of forage resources and the development of pastoral industry in the farming-pastoral zone of the Songnen Plain. *Acta Ecologica Sinica*, **26**, 601-609 (in Chinese).

Xiao, C.W., Janssens, I.A., Liu, P., Zhou, Z.Y. & Sun, O.J. 2007. Irrigation and enhanced soil carbon input effects on below-ground carbon cycling in semiarid temperate grasslands. *New Phytologist*, **174**, 835-46.

Xiao, X.P., Song, N.P., Xie, T.T. & Fang, K. 2013. Formation mechanism and community characteristics of fenced grassland in desert steppe. *Acta Prataculturae Sinica*, **22**, 321-327 (in Chinese).

Xin, L.Z. & Sai, S.B. 1990. Dynamics of primary productivity of desert steppe in Inner Mongolia. *Grassland of China*, 40-46 (in Chinese).

Xing, Q., Liu, D.S. & Cong, Z.J. 1988. Preliminary study on the dynamic of the rangeland productivity in Inner Mongolia. *Prataculture of Inner Mongolia*, 26-43 (in Chinese).

Xing, Q., Liu, Y.Z. & Han, Z.M. 1994. Dynamics of aboveground biomass and nutrition of typical steppe in Inner Mongolia. *Grassland of China*, 34-38 (in Chinese).

Xiong, D.P., Shi, P.L., Sun, Y.L., Wu, J.S. & Zhang, X.Z. 2014. Effects of Grazing Exclusion on Plant Productivity and Soil Carbon, Nitrogen Storage in Alpine Meadows in Northern Tibet, China. *Chinese Geographical Science*, **24**, 488-498.

Xue, X.J. 2009. *Response of simulated climate change on the changes of soil and plant carbon and nitrogen with different altitude in alpine meadow*. Northwest institute of plateau biology, The Chinese Academy of Science, Qinghai (in Chinese).

Yan, Y., Liu, S.Z. & Zhou, W. 2006. Dynamic of Grassland Biomass in Different Degenerative Stages. *Wuhan University Journal of Natural Sciences*, **11**, 958-962.

Yan, Y., Zhang, J.G., Zhang, J.H., Fan, J.R. & Li, H.X. 2005. The belowground biomass in alpine grassland in Nakchu Prefecture of Tibet. *Acta Ecologica Sinica*, **25**, 26-31 (in Chinese).

Yang, D.L. 1989. Dynamics of primary productivity in Leymus chinense and Carex pediformis pasture. *Prataculture of Inner Mongolia*, 22-26 (in Chinese).

Yang, D.L., Zhang, M., Ge, R.L. & Hu, Q.T. 1994. Biomass dynamics of the four rangelands in Hulun Buir. *Pratacultural Science of China*, **11**, 12-16 (in Chinese).

Yang, F.T., Wang, Q.J. & Shi, S.H. 1987. The allocation of the biomass and energy in Kobresia humilis meadow in Haibei District, Qinghai Province. *Acta Phytoecologica et Geobotanica Sinica*, **11**, 106-112 (in Chinese).

Yang, H.T., Li, X.R., Wang, Z.R., Jia, R.L., Liu, L.C., Chen, Y.L., Wei, Y.P., Gao, Y.H. & Li, G. 2014a. Carbon sequestration capacity of shifting sand dune after establishing new vegetation in the Tengger Desert, northern China. *Science of the Total Environment*, **478**, 1-11.

Yang, J.P., Mi, R. & Liu, J.F. 2009. Variations in soil properties and their effect on subsurface biomass distribution in four alpine meadows of the hinterland of the Tibetan Plateau of China. *Environmental Geology*, **57**, 1881-1891.

Yang, X.X., Ren, F., Zhou, H.K. & He, J.S. 2014b. Responses of plant community biomass to nitrogen and phosphorus additions in an alpine meadow on the Qinghai-Xizang Plateau. *Chinese Journal of Plant Ecology*, **38**, 159-166 (in Chinese).

Yang, Y., Guo, K., Zhao, L.Q., Zhao, W.H., Qiao, X.G., Liu, H.M. & Liu, Z.C. 2014c. Community characteristics of Stipa roborowskyi steppe in Xizang. *Chinese Journal of Plant Ecology*, **38**, 635-639 (in Chinese).

Yang, Y.H., Fang, J.Y., Ma, W.H., Guo, D.L. & Mohammat, A. 2010. Large-scale pattern of biomass partitioning across China's grasslands. *Global Ecology and Biogeography*, **19**, 268-277.

Yu, P.J., Li, Q., Jia, H.T., Zheng, W., Wang, M.L. & Zhou, D.W. 2013. Carbon stocks and storage potential as affected by vegetation in the Songnen grassland of northeast China. *Quaternary International*, **306**, 114-120.

Zhang, C.H. & Li, J.D. 1994. Aboveground production structure and regularities of biomass formation of Aeluropus littoralisvar. sinensis community in Northeast saline meadow. *Grassland of China*, **14**, 9-13 (in Chinese).

Zhang, F., Qi, B., Wen, F., Zhang, D.G., Wu, H. & Zhang, L. 2011. Analysis of the change of carbon storage in alpine arid grassland. *Acta Prataculturae Sinica*, **20**, 11-18 (in Chinese).

Zhang, H. 1990. Primary productivity and conversion efficiency of solar energy in grassland, Yanchi, Ningxia Province. *Agricultural resource and utilization in Yanchi, Ningxia* ed. by J.H. Fu., pp. 208–214. Ningxia Renmin Press, Yinchuan (in Chinese).

Zhang, H. 1999. Dynamics of above-ground biomass and energy efficient in the grass-forb grasslands of southern Mu Us Sandland. *Pratacultural Science of China*, **16**, 9-14 (in Chinese).

Zhang, J., Zhang, Q., He, J.M. & Sun, B. 2007. Estimation and analysis of grass biomass in desert by remote sensing *Acta ecologica sinica*, **27**, 2294-2301 (in Chinese).

Zhang, J.Y., Wang, Y., Zhao, X. & Zhang, T. 2005. Grassland recovery by protection from grazing in a semi‐arid sandy region of northern China. *New Zealand Journal of Agricultural Research*, **48**, 277-284.

Zhang, L.Y. 1985. Aboveground biomass in Haloxylon persicum community. *Chinese Bulletin of Botany*, **3**, 52-54 (in Chinese).

Zhang, M.X., Sun, C.Z., Laobusheng, D. & Suo, A.M. 1992. Dynamics of aboveground biomass in Stipa krylovii grassland. *Prataculture of Inner Mongolia*, 48-51 (in Chinese).

Zhang, N. & Liang, Y.M. 1999. Comparative studies on aboveground numerical characteristics and their relationships with soil moisture of two kinds of natural grassland in loess hilly region. *Acta Botanica Boreali-Occidentalia Sinica*, **19**, 494-501 (in Chinese).

Zhang, T.H., Su, Y.Z., Cui, J.Y., Zhang, Z.H. & Chang, X.X. 2006. A Leguminous Shrub Caragana microphylla. in Semiarid Sandy Soils of North China. *Pedosphere*, **16**, 319-325.

Zhang, X.H., Zhu, J.Z., Sun, Z.J., Qi, G.L., Zheng, W. & Gu, W.R. 2014. Influence of grazing intensity on the aboveground biomass and nutrient dynamics of community. *Pratacultural Science*, **31**, 116-124 (in Chinese).

Zhao, C.Z. & Ren, H. 2012. Individual spatial pattern and spatial association of Stipa krylovii population in Alpine Degraded Grassland. *Acta Ecologica Sinica*, **32**, 6946-6954 (in Chinese).

Zhao, H.T. 2007. *Controls of carbon stocks and carbon mineralization potential in Inner Mongolia*. The Institute of Botany, The Chinese Academy of Science, Beijing (in Chinese).

Zhao, N.N., Guggenberger, G., Shibistova, O., Thao, D.T., Shi, W.J. & Li, X.G. 2014. Aspect-vegetation complex effects on biochemical characteristics and decomposability of soil organic carbon on the eastern Qinghai-Tibetan Plateau. *Plant and Soil*, **384**, 289-301.

Zhao, W., Chen, S.P., Han, X.G. & Lin, G.H. 2009. Effects of long-term grazing on the morphological and functional traits of Leymus chinensis in the semiarid grassland of Inner Mongolia, China. *Ecological Research*, **24**, 99-108.

Zhao, X.Y., Li, J.L., Liu, H. & Yang, Q.Z. 1994. Dynamics of productivity of Leymus chinense grassland in Zhaozhou County. *Pratacultural Science of China*, **11**, 21-24 (in Chinese).

Zheng, X.X., Zhao, J.M., Zhang, Y.G., Wu, Y.Q., Jin, T.T. & Liu, G.H. 2007. Variation of grassland biomass and its relationships with environmental factors in Hulunbeier, Inner Mongolia. *Chinese Journal of Ecology*, **26**, 533-538 (in Chinese).

Zhou, H.K., Tang, Y.H., Zhao, X.Q. & Zhou, L. 2006. Long-term grazing alters species composition and biomass of a shrub meadow on the Qinghai-Tibet Plateau. *Pakistan Journal of Botany*, **38**, 1055-1069.

Zhou, Y.T., Fu, G., Shen, Z.X., Zhang, X.Z., Wu, J.S., Li, Y.L. & Yang, P.W. 2013. Estimation model of aboveground biomass in the Northern Tibet Plateau based on remote sensing date. *Acta Prataculture Sinica*, **22**, 120-129 (in Chinese).

Zhu, Z.C. & Jia, D.L. 1993. Biomass of Artemisia gmelinii community in the Loess Plateau in North Shaanxi Province. *Acta Ecologica Sinica*, **13**, 243-251 (in Chinese).

Zhu, Z.C. & Jia, D.L. 1996a. Biomass of Leymus dasystachys community. *Pratacultural Science*, **13**, 3-5 (in Chinese).

Zhu, Z.C. & Jia, D.L. 1996b. Biomass of Calamagrostis pseudophragmites community. *Acta Ecologica Sinica*, **16**, 40-49 (in Chinese).

Zu, Y.G. 1991. Primary productivity of leymus chinensis rangeland in northeast china. *Bulletin of Botanical Research*, **11**, 117-122 (in Chinese).

Zuo, X.A., Zhao, H.L., Zhao, X.Y., Guo, Y.R., Yun, J.Y., Wang, S.K. & Miyasaka, T. 2009. Vegetation pattern variation, soil degradation and their relationship along a grassland desertification gradient in Horqin Sandy Land, northern China. *Environmental Geology*, **58**, 1227-1237.
